# A Bifunctional T3SS‐Effector Simultaneously Cleaves Host MAP Kinase and Inhibits PPM1A Phosphatase

**DOI:** 10.1002/advs.202509702

**Published:** 2026-03-28

**Authors:** Yaakov Socol, Lihi Gur‐Arie, Netanel Tzarum, Tamara Wellins, Naama Katsowich, Miriam Ravins, Michal Bejerano‐Sagie, Klil Cohen, Oded Livnah, Joshua N. Adkins, Ernesto Nakayasu, Alexei Savchenko, Yeu Khai Choong, Nikhil Kumar Tulsian, Saturo Machida, Sigal Ben‐Yehuda, Yael Litvak, J. Sivaraman, Ilan Rosenshine

**Affiliations:** ^1^ Department of Microbiology and Molecular Genetics Faculty of Medicine The Hebrew University of Jerusalem Jerusalem Israel; ^2^ Department of Biological Chemistry Alexander Silverman Institute of Life Sciences The Hebrew University of Jerusalem Jerusalem Israel; ^3^ The Wolfson Center for Applied Structural Biology The Edmond J. Safra Campus The Hebrew University of Jerusalem Jerusalem Israel; ^4^ Biological Sciences Division Pacific Northwest National Laboratory Richland Washington USA; ^5^ Department of Microbiology Immunology and Infectious Diseases University of Calgary Calgary Alberta Canada; ^6^ Department of Biological Sciences National University of Singapore Singapore Singapore; ^7^ Department of Biochemistry National University of Singapore Singapore Singapore

**Keywords:** enteropathogenic *E. coli*, JNK, MAPK, NleD, NleE, p38, PPM1A

## Abstract

NleD belongs to a family of metalloproteases produced by multiple pathogens and functions as a Type III secretion system (T3SS) effector. NleD inactivates the p38 and JNK MAP kinases by cleaving them at a single site within the conserved threonine‐X‐tyrosine (TXY) motif. Here, we show that NleD from enteropathogenic *E. coli* (EPEC) interacts with PPM1A, a host metallophosphatase that targets multiple substrates, including the MAPK TXY motif. Binding of NleD inhibits the phosphatase activity of PPM1A while preserving NleD's proteolytic function. Structural analysis of the NleD‐PPM1A complex reveals that NleD suppresses PPM1A activity by blocking phospho‐protein substrates from accessing its catalytic pocket. Intriguingly, using a *Citrobacter rodentium* murine infection model, we found that NleD can enhance intestinal colonization in a manner independent of its protease activity, possibly via interaction with PPM1A. Together, these findings identify NleD as a bifunctional effector, highlighting the sophisticated strategies by which T3SS effectors manipulate key host signaling pathways.

## Graphical abstract

Pathogenic bacteria exploit the metalloprotease effector NleD to subvert host defenses. Structural, biochemical, and infection analyses reveal a bifunctional mechanism by which NleD binds and inhibits the host phosphatase PPM1A while preserving its proteolytic activity against MAPKs. This dual strategy enables precise modulation of host signaling pathways, ultimately promoting bacterial survival and colonization.

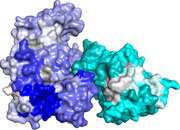

## 1 Introduction

Following pathogen infection, bacterial components activate signaling cascades that induce the host inflammatory response. The mitogen‐activated protein kinase (MAPK) signaling pathway is conserved in all eukaryotes and is involved in fundamental cell processes, including a pivotal role in inflammation signaling [1, 2]. MAPKs are serine/threonine kinases that can be divided into three main subfamilies, each including several isoforms; the Jun N‐terminal Kinase (JNK), the Extracellular signal‐Regulated Kinase (ERK), and p38 [1]. MAPKs are activated following diverse stimuli, such as sensing of bacterial components or cytokines such as TNFα and IL‐1 [2]. In other cases, the MAPK cascade is initiated in response to specific intracellular signals, such as the stress induced by the translation inhibitor anisomycin [1]. The MAPK cascade transduces the signal through three tiers of protein kinases. The first tier includes MAPK kinase kinases (MAP3K), such as TAK1 and MLK7, which phosphorylate the downstream tier of MAPK kinases (MAP2K). Consequently, MAP2Ks phosphorylate adjacent threonine (T) and tyrosine (Y) residues at a conserved TXY motif of MAPKs, where X is either proline, glycine, or glutamic acid [1]. TXY phosphorylation leads to activation of the MAPKs, which then phosphorylate downstream proteins, including other kinases and transcription or translation factors [1, 2].

The MAPK signaling is negatively regulated by protein phosphatases, such as metal‐dependent protein phosphatases 1A and 1B (PPM1A, PPM1B) and other PPM family members [3]. This family comprises serine/threonine protein phosphatases, which share a conserved catalytic domain and have various substrates, frequently shared by several phosphatases [3]. The PPM1A substrates include the stress‐activated kinases (e.g., p38, JNK, TAK1), placing it as a negative regulator of stress responses [3]. PPM1A/B also terminates TGF‐β signaling by dephosphorylating SMAD2/3 [4], and acts on CDK substrates, influencing cell cycle progression [5, 6, 7]. PPM1A also downregulates inflammation via dephosphorylation of TBK1 [8], MAVS [8], RelA, and IKKβ [9], and it promotes tissue repair and regeneration via dephosphorylation of YAP/TAZ (hippo signaling) [10] and p62/SQSTM1 (autophagy receptor) [11]. PPM1A and PPM1B undergo irreversible N‐myristoylation, where the myristoyl group is predicted to be located near the opening of PPM1A catalytic pocket [12], thereby enhancing its substrate‐specificity [12]. The N‐myristoylation involves elimination of the first methionine and ligation of a 14‐carbon saturated fatty myristic acid to a glycine residue at position 2 (G2) [12].

**FIGURE 1:**
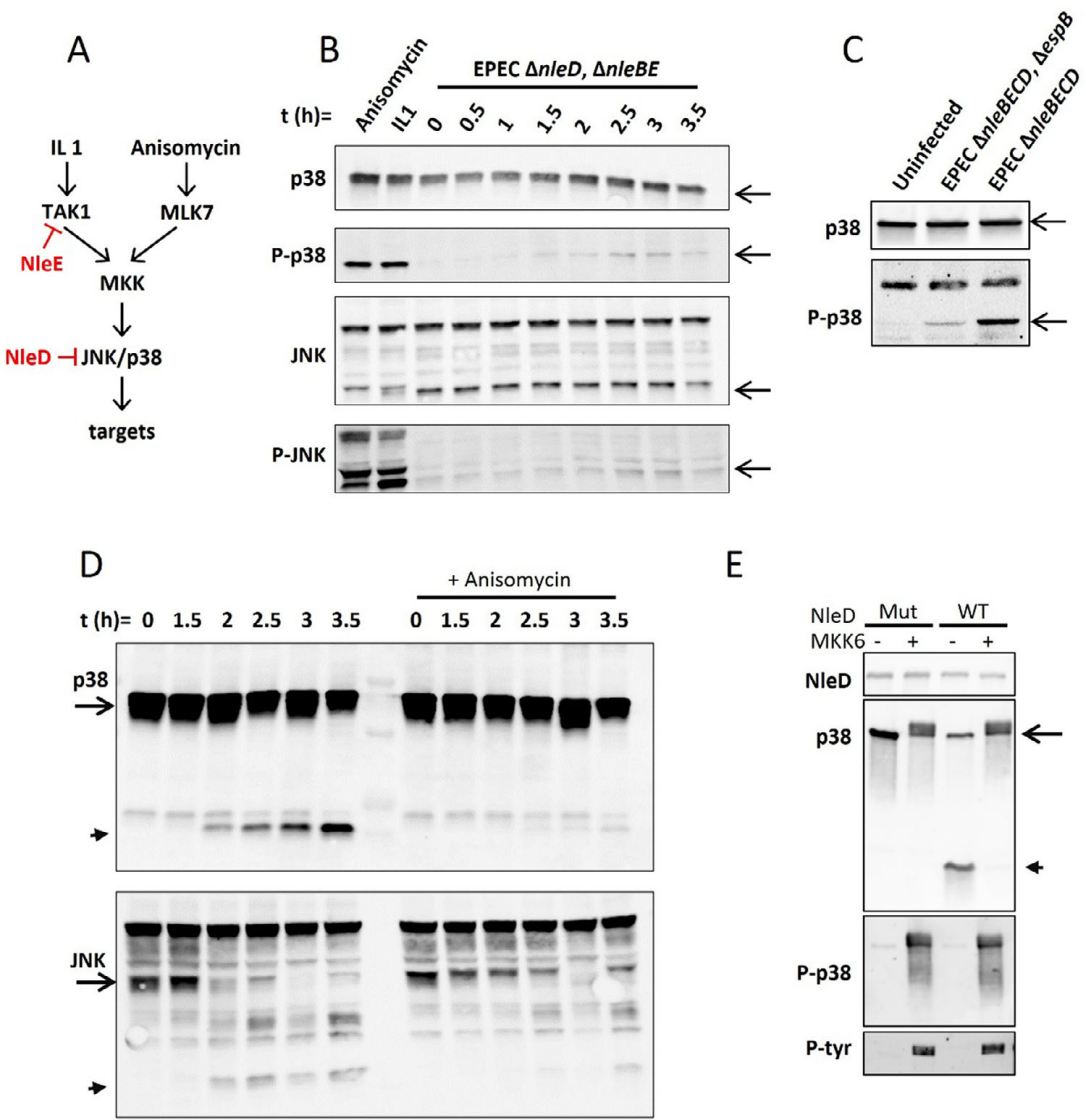
Activated p38 and JNK are resistant to cleavage by NleD. (A) Schematic diagram of the MAPK signaling pathway induced by IL‐1 and anisomycin and EPEC effectors that intercept this signaling. The arrows indicate the activation of phosphorylation of the respective protein. The inhibiting effectors are indicated in red fonts. (B) HeLa cells were infected with EPEC Δ*nleD*, Δ*nleBE*, and sampled at different time points. Extracted host proteins were analyzed by Western blot using anti‐p38, anti‐phospho‐p38 (P‐p38), anti‐JNK, and anti‐phospho‐JNK (P‐JNK), indicated by arrows. Induction of MAPK phosphorylation with anisomycin and IL‐1 was used as a positive control. (C) HeLa cells were left uninfected or infected for 3 h with EPEC deleted of the *nleB*, *nleE, nleC*, and *nleD* T3SS effector genes (Δ*nleBECD*), or with the same mutant additionally deleted for *espB*, which encodes an essential component of the T3SS (Δ*nleBECD*, Δ*espB*). Following infection, host cell proteins were extracted and analyzed by Western blot using anti‐p38 and anti‐phospho p38 (P‐p38) antibodies. (D) HeLa cells were treated with anisomycin or remained untreated. This was followed by infection with EPEC. Proteins were then extracted at the indicated time point and subjected to Western blot analysis with anti‐JNK and anti‐p38 antibodies to detect JNK and p38 cleavage. Intact proteins and cleavage products are indicated by an arrow and an arrowhead, respectively. The reason for the reduced JNK levels at 3 h post‐infection is unknown. (E) Purified NleD‐SBP tagged (WT) or corresponding NleD E143A mutant (Mut) were incubated with purified recombinant p38. For purification, p38 was either expressed alone or co‐expressed with constitutively active MKK6 that phosphorylates p38. Intact and degraded p38 were detected using anti‐p38, anti‐phosphotyrosine (P‐tyr) and anti‐phospho‐p38 antibodies. Arrows point to full‐length proteins and arrowheads to cleavage products. Western blots in B, C, D, and E were normalized by adjusting total protein concentrations and validated by the Stain Free gels (Bio‐Rad). All the experiments were repeated at least three times.

Many Gram‐negative pathogens, including enteropathogenic *E. coli* (EPEC), use a Type III Secretion System (T3SS) molecular syringe to deliver proteins termed effectors into the host cells [13]. The injected effectors target different host‐cell processes and signaling cascades to allow efficient EPEC colonization [13]. Host cells possess an intrinsic ability to detect the T3SS of EPEC and, in response, activate the NF‐κB signaling pathway [14]. However, the bacteria inject effectors such as NleC and NleE that intercept the T3SS sensing and NF‐κB signaling (Figure 1A) [14]. NleC cleaves NF‐κB proteins [15, 16], whereas NleE blocks the activation of the MAP3K TAK1 and thus is expected to block both NF‐κB and MAPK signaling [17, 18, 19]. Concomitantly, the effector NleD blocks MAPK signaling via the direct cleavage of JNK and p38 [8].

Here we show that NleD cleaves only the inactive, un‐phosphorylated MAPKs, and is incapable of cleaving their activated phospho‐state. We further found that the injected NleD of EPEC (NleD_EPEC_) binds specifically to N‐myristoylated PPM1A. Notably, whereas NleD cleaves the MAPKs activation loop between the X and Y residues of the TXY motif, PPM1A targets and dephosphorylates both the phosphothreonine and phosphotyrosine residues within this motif. Biochemical and structural analysis of the NleD‐PPM1A complex shows that the bound NleD masks the PPM1 catalytic site entrance, thereby inhibiting it. Experimental mouse infection with *Citrobacter* *rodentium* expressing different NleD variants suggests that both JNK/p38 cleavage and inhibition of PPM1A by NleD significantly contribute to the pathogen's virulence. Our results reveal novel aspects of T3SS effectors’ mechanisms, exposing their dual‐function potential.

## 2 Results

### 2.1 Sensing of EPEC by the Host Leads to MAPK Signaling Activation

Host cells can detect the insertion of the translocon channel of the EPEC T3SS into its membrane, leading to activation of the TAK1‐NF‐κB signaling [14]. Since TAK1 is also a MAP3K, we predicted that T3SS‐sensing should also lead to p38 and JNK activation. To test this prediction, we infected host cells with EPEC lacking the NleE and NleD effectors known to intercept the TAK1‐MAPK signaling [15, 18, 20], as reconfirmed here (Figure 1A; Figure S1). We then sampled the infected cells at time points up to 3.5 h post‐infection and analyzed the levels of total and phosphorylated p38 and JNK (P‐p38 and P‐JNK). Notably, under these conditions, the bacteria produce functional T3SS only around 2 h post‐infection [21]. A moderate and T3SS‐dependent increase in the levels of P‐p38 and P‐JNK was observed as early as 2 h after infection, subsequently peaking at 3 h (Figure 1B,C). These results confirmed that MAPK signaling is activated upon T3SS sensing.

### 2.2 Phosphorylated p38 and JNK Are Resistant to Cleavage by NleD

Given that the MAPK phosphorylation site and the NleD cleavage site overlap (i.e., both are at the TXY motif) [1, 15], we asked whether NleD can cleave phosphorylated p38 and JNK. To this end, we used EPEC to infect host cells pre‐treated with anisomycin, which does not affect EPEC growth, and boosts p38/JNK phosphorylation (Figure 1A; Figure S1) [22]. We noted a marked decrease in the efficiency of JNK and p38 cleavage in anisomycin‐treated cells, hinting that phosphorylated MAPKs are less sensitive to cleavage by NleD (Figure 1D). To further test whether phosphorylated p38 and JNK are more resistant to NleD, we generated plasmids expressing JNK and p38 phospho‐mimicry mutants where the threonine and tyrosine residues in the TXY motif were replaced by AXF, EXY, TXD, and EXD. We next tested the capacity of NleD to cleave these mutants by co‐expressing each of them with NleD in *E. coli* BL21. NleD could efficiently cleave p38 containing AGF or EGY instead of TGY, or JNK containing APF or EPY, but failed to cleave the corresponding TGD, EGD, or TPD mutants (Figure S2). Taken together, these findings indicate that phosphorylated p38 and JNK might be resistant to cleavage by NleD and suggest that phosphorylation of the tyrosine residue of the TXY motif is sufficient to inhibit cleavage by NleD.

To directly test if NleD can cleave phospho‐p38, we set up an in vitro system composed of NleD or a catalytically dead NleD mutant (NleD E143A), incubated with purified p38 or P‐p38. To obtain phosphorylated p38, we co‐expressed in *E. coli* p38 and constitutively active MKK6, which is the p38 cognate MAP2K [23]. Alternatively, the purified p38 was phosphorylated in vitro using purified MKK6. We then analyzed the cleavage products and phosphorylation levels using anti‐p38 (total p38), anti‐phospho‐p38 (to detect dually‐phosphorylated p38), and anti‐phospho‐tyrosine antibodies (to detect tyrosine‐phosphorylated p38). We found that NleD cleaved p38 but not phosphorylated p38 (Figure 1E; Figure S3). Taken together, these results show that phosphorylation of the TXY motif interferes with NleD activity, demonstrating that NleD specifically targets the un‐phosphorylated forms of p38 and JNK.

### 2.3 NleD Binds PPM1A, a Phosphatase That Targets p38 and JNK

**FIGURE 2:**
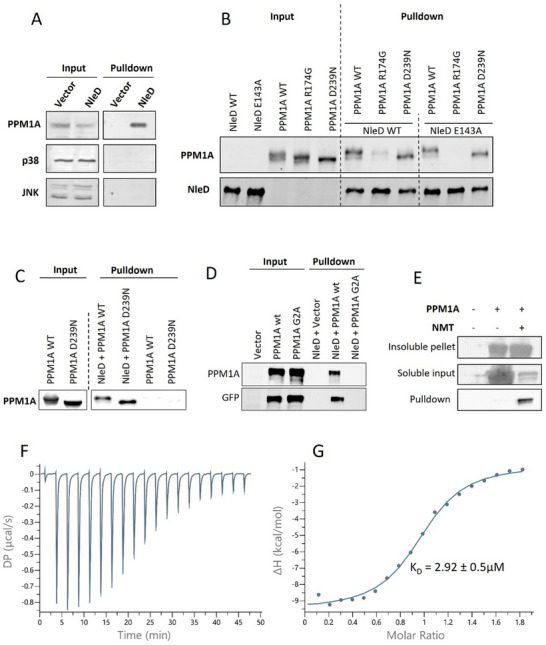
NleD binds N‐myristoylated PPM1A. (A) Proteins from *E. coli* containing a plasmid expressing SBP‐tagged wild‐type NleD (NleD), or an empty plasmid (Vector), were extracted, immobilized on streptavidin beads, and subsequently incubated with lysate of HeLa cells. Bound proteins were analyzed by Western blot with antibodies against p38, JNK, and PPM1A, as indicated. (B) SBP‐tagged wild‐type NleD (NleD WT) or catalytically dead NleD_E143A_ mutant were immobilized on streptavidin beads and subsequently incubated with lysate of HEK293T cells transfected with plasmids expressing wild‐type or mutated PPM1A_R174G_ and PPM1A_D239N_. Bound proteins were analyzed by Western blot with antibodies against SBP (to detect NleD), or PPM1A, as indicated. (C) Lysate of HEK293T cells transfected with plasmids expressing wild‐type PPM1A (PPM1A WT), or mutated PPM1A (PPM1A D239N) were incubated with streptavidin beads loaded, or not, with SBP‐NleD. The capacity of PPM1A to bind to the beads was tested by Western blot using an anti‐PPM1A antibody. (D) SBP‐tagged NleD_EPEC_ immobilized on streptavidin beads was incubated with the lysate of HEK293T cells transfected with plasmids expressing PPM1A fused to GFP, or the PPM1A‐G2A mutant fused to GFP. Proteins were analyzed by Western blot with antibodies against GFP or PPM1A as indicated. Beads loaded with lysate of *E. coli* that do not express NleD (Vector) were used as a negative control. (E) PPM1A was expressed in *E. coli* BL21 with or without co‐expression of human myristoyl transferase (NMT). The bacterial lysate was then added to SBP‐NleD immobilized on streptavidin beads. Bound proteins were extracted and subjected to Western blot analysis using an anti‐PPM1A antibody. (F,G) Isothermal titration calorimetry analysis of PPM1A interacting with NleD. The left panel (E) shows the raw ITC data for the injection of PPM1A protein into the sample cell containing NleD. (F) Solid dots indicate the experimental data, and the best fit was obtained from a nonlinear least squares method, using a single‐site binding model depicted by a continuous line. The titration of PPM1A against NleD had a K_D_ of 2.92 ± 0.5 µm. Results of one experiment out of four with similar results is shown. Western blots were normalized when applicable by adjusting total protein concentrations and validated by the Stain Free gels (Bio‐Rad). All the experiments were repeated at least three times.

To better understand the NleD function, we used mass spectrometry (MS) to identify interacting partners of NleD in extracts of HEK293T host cells. Immobilized NleD, N‐terminally fused to GST, was incubated with an extract of HEK293T cells, and the captured proteins were subjected to MS analysis. As a control, we performed parallel analysis using GST‐NleE. Notably, the MS analysis failed to identify the known NleD substrates p38 and JNK, presumably due to the very transient nature of the enzyme‐substrate interaction (Table 1 and Data S1). Nevertheless, NleD exhibited robust binding to protein metallophosphatase 1A and 1B (PPM1A and PPM1B, respectively). Intriguingly, NleD and PPM1A share the same substrates: the TXY motif of p38 and JNK. NleD cleaves the X─Y bond [15], while PPM1A dephosphorylates the T [3]. To verify the NleD‐PPM1A interaction, we repeated the co‐purification employing NleD N‐terminally tagged with streptavidin‐binding peptide (SBP‐NleD). Western blot analysis confirmed that NleD specifically binds to the native PPM1A (Figure 2A). Furthermore, the NleD‐bound PPM1A appears intact; thus, in contrast to p38 and JNK, PPM1A is most likely not a substrate of NleD protease activity. Similarly to the MS results, western blot analysis failed to identify the NleD substrates, p38 and JNK, as NleD binding partners.

**TABLE 1:** Major identified interacting partners of NleD and NleE. Data were obtained from two independent experiments, and the number of unique peptides from each experiment is shown together with an average LFQ intensity score.

Protein name	Gene name	Unique peptides NleD	Unique peptides NleE	LFQ intensity NleD	LFQ intensity NleE
Protein phosphatase 1A	PPM1A	17/18	0/0	4.58 × 10^7^	0
Protein phosphatase 1B	PPM1B	14/15	0/0	2.86 × 10^7^	0
Coatomer subunit beta	COPB2;Copb2	3/1	26/26	1.39 × 10^5^	2.54 × 10^7^
Coatomer subunit epsilon	COPE	1/0	9/9	0	1.79 × 10^7^
Dynactin subunit 4	DCTN4	0/1	4/4	0	8.77 × 10^6^

### 2.4 Catalytically Dead Mutants of PPM1A and NleD Still Interact

We next tested whether the catalytic activity of PPM1A or that of NleD is required for their interaction. We examined the capacity of recombinant NleD immobilized on beads to bind to two reported inactive mutants of PPM1A, ectopically expressed in HEK293T cells. We found that mutating the arginine residue in position 174 of PPM1A (R174G) drastically diminished the NleD‐PPM1A interaction (Figure 2B). Of note, this residue is reported to form salt bridges that stabilize the structure of the catalytic domain of PPM1A [24]. In contrast, a PPM1A mutant in the aspartic acid residue at position 239 (D239N), which is also required for catalytic activity but lacks the structural significance of the former variant, still binds NleD similarly to the wild‐type PPM1A (Figure 2B,C). Likewise, the catalytically dead NleD E143A [9, 25], efficiently binds wild‐type PPM1A and the PPM1A D239N mutant (Figure 2B). These results demonstrate that the catalytic activities of NleD and PPM1A are not required for their physical interaction.

### 2.5 NleD Specifically Binds to Myristoylated PPM1A

Our attempts to verify the NleD‐PPM1A interaction using recombinant PPM1A produced in *E. coli* BL21 were unsuccessful, implying that a post‐translational modification of PPM1A might be required for the interaction with NleD. PPM1A undergoes N‐myristoylation [12], and we thus tested NleD binding to PPM1A where the myristoylated residue, glycine at position 2, was replaced by alanine (PPM1A_G2A_). We transiently expressed PPM1A, or PPM1A_G2A_, fused to GFP in HEK293T cells, extracted the proteins, and used NleD to capture binding partners. The results show that NleD binds to PPM1A‐GFP but not PPM1A_G2A_‐GFP (Figure 2D), suggesting that NleD specifically binds to N‐myristoylated PPM1A. To substantiate this notion, we engineered *E. coli* to co‐express PPM1A and the human N‐myristoyltransferase (NMT), which catalyzes PPM1A myristoylation [12]. Co‐expression with NMT resulted in a marked reduction of the solubility of PPM1A, presumably due to the strong hydrophobic properties of the myristoyl group. However, co‐expression with NMT rescued the ability of PPM1A to bind NleD, reinforcing the view that NleD specifically interacts with N‐myristoylated PPM1A (Figure 2E). To assess the binding efficiency, we purified from *E. coli* BL21 His‐tagged NleD and N‐myristoylated PPM1A, and used them for isothermal titration calorimetry (ITC) experiments. The titration of PPM1A against NleD had a K_D_ of 2.92 ± 0.5 µm, with a 1:1 binding stoichiometry (Figure 2F,G). Taken together, our results with purified components show that NleD robustly and directly binds to N‐myristoylated PPM1A.

### 2.6 PPM1A dephosphorylates P‐p38, Priming It to be Cleaved by NleD

**FIGURE 3:**
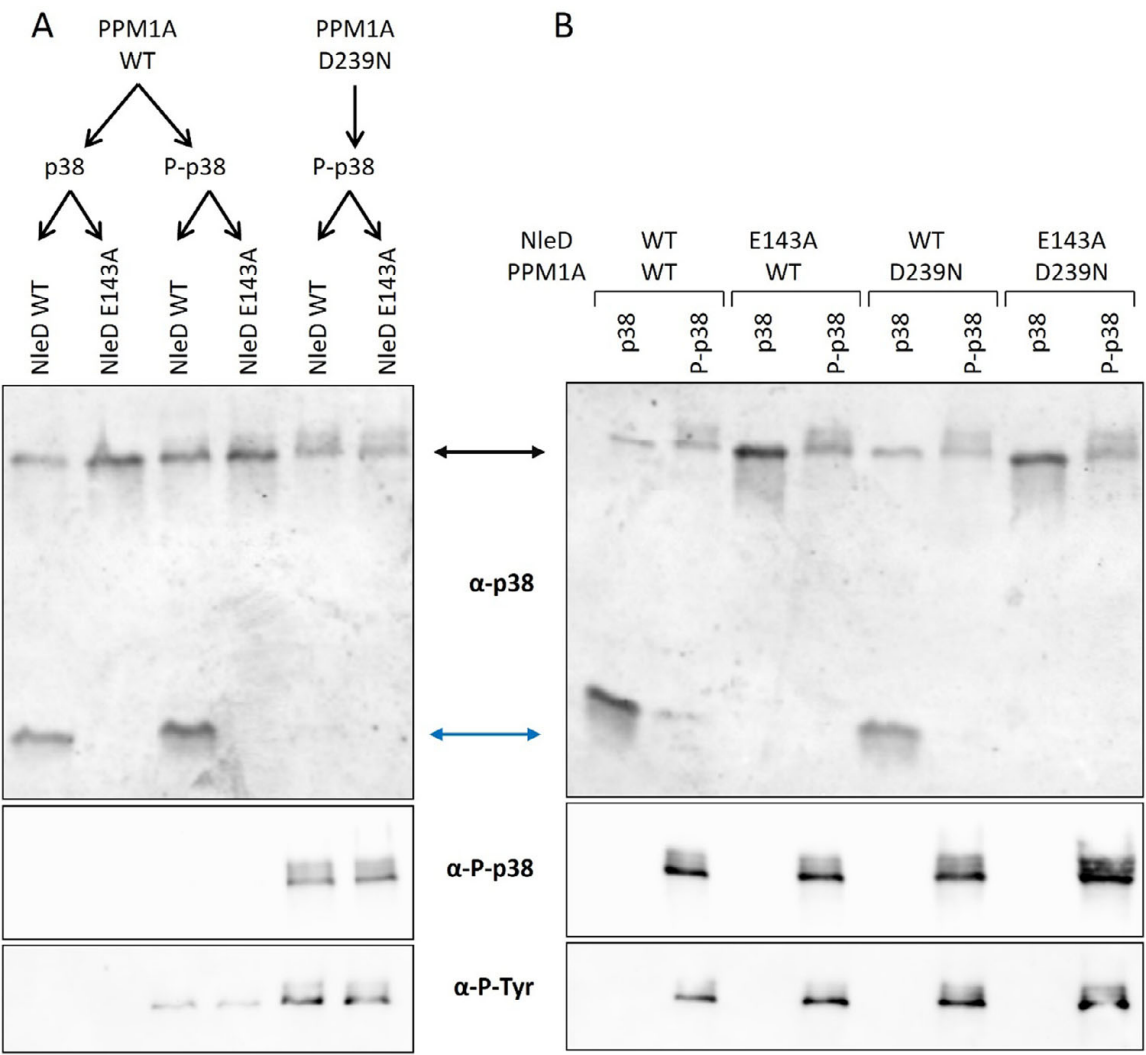
NleD inhibits dephosphorylation of the TXY motif by the bound PPM1A (A) Wild type PPM1A or the inactive mutant PPM1A_D239N_ were incubated for 1 h with p38 or phospho‐p38 (P‐p38) as indicated. Then, NleD, or catalytically dead NleD_E143A_, was added to the reaction mix, followed by incubation for an additional hour. p38 dephosphorylation and p38 cleavage were assessed using Western blot and antibodies against p38, phospho‐p38, and phospho‐tyrosine. Black and blue arrows point to intact p38 and cleavage products, respectively. (B) p38 or phospho‐p38 (P‐p38) were treated for 1 h with different NleD‐PPM1A pre‐formed complexes, including all combinations of wild‐type and mutated, catalytically inactive NleD and PPM1A (NleD_E143A_ and PPM1A_D239N_, respectively) as indicated above the lanes. Then, dephosphorylation and cleavage of p38 and phospho‐p38 were assessed using Western blot and antibodies against p38, phospho‐p38, and phospho‐tyrosine. Black and blue arrows point to intact p38 and cleavage products, respectively.

Given the inability of NleD to cleave phospho‐p38/JNK and its interaction with PPM1A, we speculated that PPM1A and NleD collaborate through initial dephosphorylation of p38/JNK by PPM1A, priming them for the subsequent cleavage by NleD. To test this premise, we examined whether the inherent activity of recombinant PPM1A purified from HEK293T cells is sufficient to dephosphorylate the TGY motif of p38. Indeed, our data show that incubation of purified PPM1A with P‐p38 resulted in dephosphorylation of the p38 TGY motif. Notably, despite being considered a serine/threonine phosphatase, our data show that PPM1A also dephosphorylated the tyrosine residues of the motif. Moreover, the dephosphorylation of p38 by PPM1A enabled its cleavage by NleD, which was subsequently added to the reaction (Figure 3A). Collectively, these results show that when P‐p38 was treated sequentially by PPM1A and then by NleD, it was dephosphorylated and then cleaved.

### 2.7 NleD Inhibits the Dephosphorylation of Phospho‐p38 by PPM1A

We next aimed at testing the coinciding activity of NleD and PPM1A when in complex. To avoid interference between their activities, we also used complexes containing catalytically dead forms of PPM1A (PPM1A_D239N_) and NleD (NleD_E143A_), which are proficient in complex formation (Figure 2B). We incubated SBP‐NleD or SBP‐NleD_E143A_ with purified PPM1A or PPM1A_D239N_ expressed in HEK293 cells, such that four different purified complexes were formed: PPM1A‐NleD, PPM1A_D239N_‐NleD, PPM1A‐NleD_E143A_, and PPM1A_D239N_‐NleD_E143A_. We then mixed each of these complexes with either p38 or P‐p38 and tested the capacity of these complexes to dephosphorylate and cleave the added P‐p38. The results show that NleD remains active when in complex, cleaving the unphosphorylated p38, but not the phosphorylated form (Figure 3B). Surprisingly, however, when associated with NleD, the bound PPM1A fails to dephosphorylate P‐p38 and to prime it for cleavage by NleD (Figure 3B). Similar results were obtained when using PPM1A in complex with the catalytically inactive NleD_E143A_, indicating that the catalytic activity of NleD is not responsible for inhibiting the capacity of PPM1A to dephosphorylate P‐p38 (Figure 3B). These results indicate that bound NleD inhibits the capacity of PPM1A to dephosphorylate p38. Since the proteolytic activity of NleD was dispensable for PPM1A inhibition, a plausible mechanism for the effect of the bound NleD is that it might interfere with P‐p38 recognition by PPM1A. In contrast, the bound PPM1A does not interfere with the proteolytic activity of NleD.

### 2.8 NleD Binds Proximal to the PPM1A Catalytic Site

**FIGURE 4:**
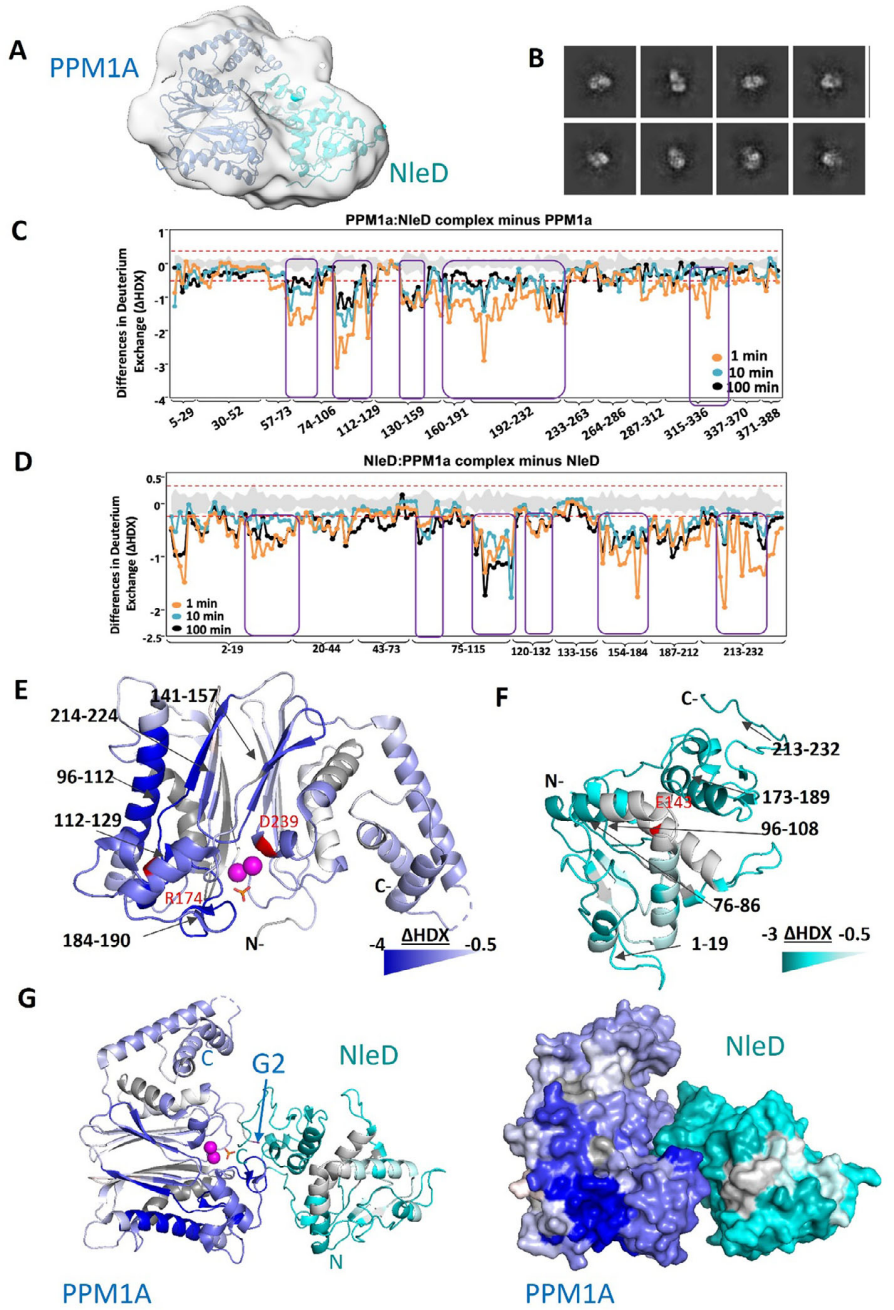
Model of the NleD‐PPM1A complex. (A) In silico model of the PPM1A (blue)‐NleD (cyan) complex fit in the experimental cryo‐EM map. (B) 2D averages selected to reconstruct the map in (A). 160 000 X magnification, 0.76 Å/pix, box size 320 pix. (C,D) Plots of differences in deuterium exchange (*y*‐axis) between the PPM1A‐NleD complex and apo states of PPM1A (panel C) and free NleD (panel D) across various peptides (*x*‐axis) at indicated deuterium labeling times. For both NleD and PPM1A, ∼96% coverage was observed, with only a few amino acids (mostly at the N‐terminus) undetected. Protein and peptide confidence levels were determined based on criteria such as minimum intensity, detection across all replicates and conditions, MS–MS fragmentation, high signal‐to‐noise ratio, and redundancy (multiple peptides detecting the same amino acid). Different peptides are grouped in clusters with their residue numbers indicated and tabulated in Table S6. Negative differences indicate protection against deuterium uptake in the PPM1A‐NleD complex compared to their free states. A significance of ± 0.3 D (99% confidence, determined using Deuteros 2.0) was considered significant and indicated by red‐dashed lines. Average values from technical triplicates were used to generate the plots, with standard deviations in gray. The regions showing the most significant changes are highlighted in purple boxes. (E,F) Differences in deuterium exchange at 1 min labeling were mapped onto PPM1A (PDB: 4RA2) shown in cartoon representation (panel E) and onto a model of NleD predicted by the Robetta server (panel F) are indicated by the color scheme. Key residues PPM1A:R174, D239, and NleD:E143 are highlighted in red. (G) The model of the PPM1A‐NleD complex generated from the ClusPro server is shown with the HDX differences overlaid. HDX differences in PPM1A are colored in shades of blue, while those on NleD are in shades of cyan. Greater protection against deuterium exchange for the two proteins is predominantly centered around their interaction interfaces. The left and right panels are the same model in cartoon and surface representations, respectively. The arrow points to the myristoylation site PPM1A:2G in the cartoon model.

To elucidate how NleD inhibits PPM1A, we performed in silico structure prediction to dock the theoretical model of NleD to the known PPM1A structure using the ClusPro server [26]. In addition, cryo‐EM data were collected for the N‐myristoylated PPM1A‐NleD complex. Template‐free 2D classification and 3D reconstruction identified a spindle‐shaped complex. The predicted model fits well with the shape of the 5.3 Å resolution cryo‐EM map, revealing the NleD‐PPM1A interacting regions (Figure 4A,B; Figure S4). Next, we mapped the NleD‐PPM1A interactions by hydrogen‐deuterium exchange mass spectrometry (HDXMS) [27, 28]. For this analysis, NleD, PPM1A, and the NleD‐PPM1A complex were placed in a D_2_O‐rich buffer, allowing the hydrogen atoms in the proteins to exchange with deuterium atoms from the solution. A slower rate of deuterium incorporation to a specific residue indicates lower accessibility of hydrogen atoms in this residue to the solution. Residues in the contact surface of the bound proteins are expected to be sequestered from the environment and thus should incorporate deuterium to a lesser extent when in complex than the same residues when measured as separate proteins. Mapping of deuterium changes (Figure 4C,D), onto the high‐resolution crystal structure of PPM1A showed that PPM1A residues, located proximally to the catalytic pocket, exhibited significantly reduced exchange rate when in complex with NleD, supporting the notion that NleD might mask the PPM1A active site (Figure 4C–F; Figure S5A). Notably, the interacting regions observed in the in silico/EM model are consistent with the HDX findings. Moreover, the binding of NleD reduced the protein‐wide conformational dynamics of PPM1A, which includes the key residue Arg174 (in peptide 172–183) and Asp239 (in peptide 233–243) (Figure 4C,E; Figure S5A), as corroborated with our mutational analyses (Figure 2B). Likewise, we mapped the NleD residues that were protected by PPM1A against deuterium exchange, onto a model of NleD (Figure 4D,F; Figure S5B). In addition to the C‐terminal tail of NleD, large‐magnitude differences in deuterium exchange were observed across residues 76–86 and 96–108 in PPM1A‐bound NleD (Figure 4D,F; Figure S5B), suggesting its interactions with PPM1A. The engagement of the NleD C‐terminal region in proximity to the catalytic site of PPM1A is reflected in our model (Figure 4G). The HDXMS results indicate that the residues near the PPM1A catalytic site are involved in the interaction, supporting the notion that within the complex, the catalytic site of PPM1A is blocked while that of NleD remains free, exhibiting a high deuterium exchange rate (Figure 4G; Figure S5A).

Collectively, combining the *in‐silico* modeling of the complex with the results from HDXMS interaction maps, and from a low‐resolution (5.3 Å) cryo‐EM map, a model for the PPM1A‐NleD complex was generated (Figure 4G). This model shows that NleD binds and restricts access to the catalytic pocket of PPM1A, thereby preventing its activity. The access to the Asp239 of PPM1A, a catalytic residue for PPM1A activity, is hindered by the C‐terminal region of NleD, in accordance with the inactivity of PPM1A in the presence of NleD.

### 2.9 PPM1A in Complex with NleD Hydrolyzes Small Substrates

**FIGURE 5:**
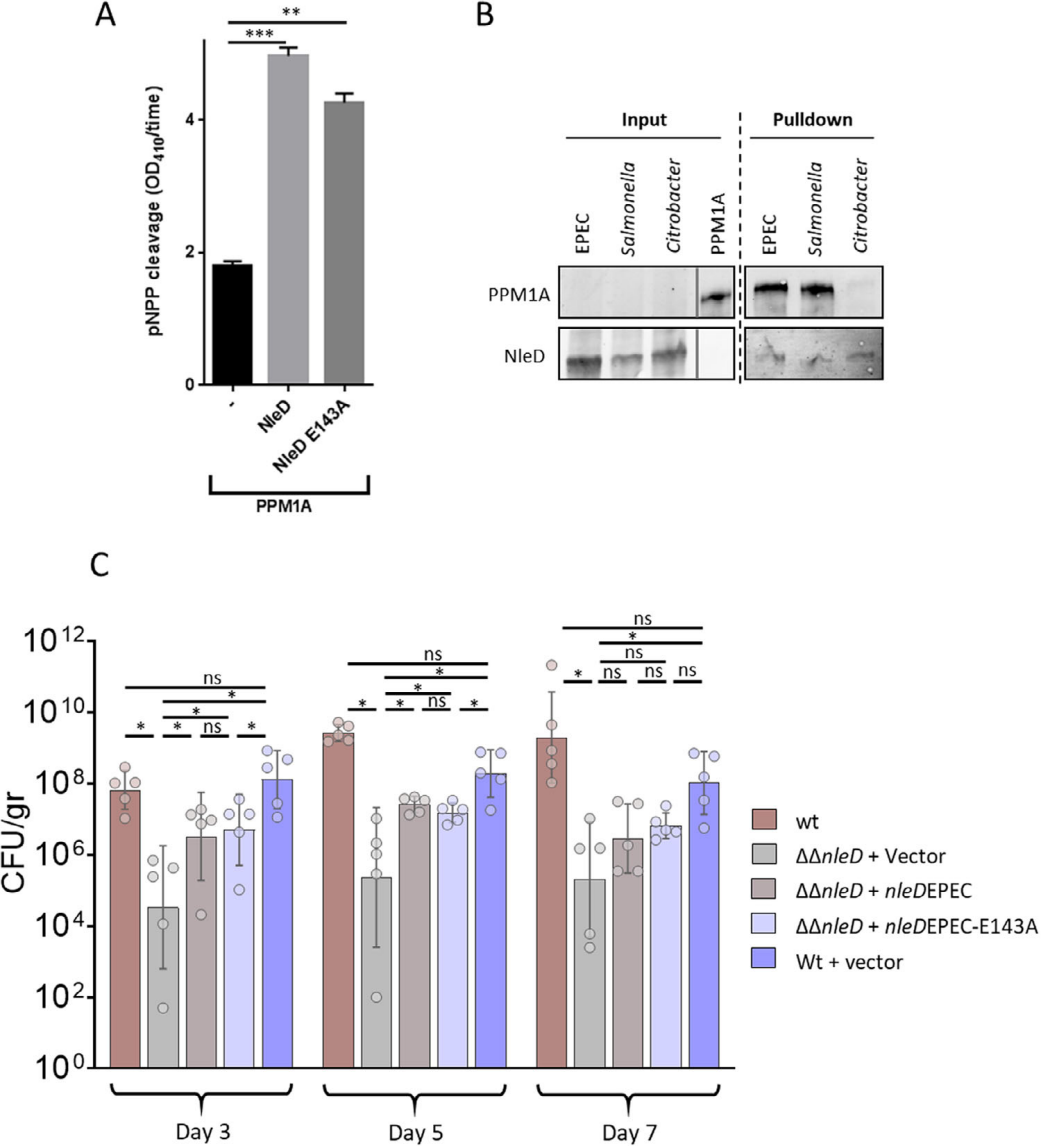
Validation of NleD‐PPM1A complex formation, and role in host infection. (A) The colorimetric phosphatase substrate pNPP was treated with PPM1A or PPM1A in complex with wild‐type NleD or the NleD_E143A_ mutant as indicated. Hydrolysis of pNPP was assessed using absorbance at 410 nm. The rate of pNPP cleavage, represented by the slope of OD_410_ over time, is shown. Statistical significance was assessed using an unpaired *t*‐test; ^**^ *p* < 0.01, ^***^ *p* < 0.005 (*n* = 2). (B) SBP‐tagged NleDs of EPEC, *Salmonella arizonae*, or *C. rodentium* were immobilized on streptavidin beads and incubated with purified PPM1A‐FLAG. Proteins were then extracted from the washed beads and subjected to Western blot analysis using anti‐PPM1A and anti‐SBP (to detect SPB‐NleD). (C) NleD_EPEC‐E143A_ partially restores host colonization by *C. rodentium* lacking its native NleDs. Bacterial load measurement as CFU per gram feces collected at different time points after infection of mice (*n* = 5, per tested strain). Infection was carried out using the following *C. rodentium* strains: wild type (Wt); ΔΔ*nleD* complemented with empty vector (ΔΔ*nleD* + vector); ΔΔ*nleD* complemented with *nleD*_EPEC_ expressing vector (ΔΔ*nleD* + *nleD*_EPEC_); ΔΔ*nleD* complemented with NleD_EPEC‐E143A_ expressing vector (ΔΔ*nleD* + *nleD*_EPEC‐E143A_); and WT complemented with empty vector (WT + vector). All vectors are replication‐deficient and are designed to integrate into the genome, such that recombinant NleDs expression is driven by the promoter of the LEE6 operon. Bars represent geometric means ± standard error. Statistical significance was assessed using an unpaired *t*‐test; ^*^ *p* < 0.05. ns, not significant.

The model of the NleD‐PPM1A complex suggests that the restricted access to the PPM1A catalytic site depends on the size of the substrate. While access to large substrates such as P‐p38 protein is denied, small substrates such as p‐nitrophenyl phosphate (pNPP, molar mass 0.219 kD) might diffuse into its catalytic site. To test this prediction, we incubated the NleD‐PPM1A complex with pNPP. We found that in this case, NleD did not inhibit, but enhanced, the PPM1A activity (Figure 5A), exhibiting almost a three‐fold increase in pNPP hydrolysis. Similar results were observed when a catalytically inactive form of NleD was used. These results reinforce the premise that, as predicted by the model, NleD prevents the dephosphorylation of p38 by masking the catalytic site of PPM1A while the catalytic capacity remains intact.

### 2.10 NleD Homologs Differ in Their Ability to Bind PPM1A

The NleDs of EPEC and the mouse pathogen *Citrobacter rodentium* (CR) share nearly 75% sequence identity, with NleD_EPEC_ being three residues shorter (Figure S6A). Structural alignment of the predicted models of EPEC and CR NleDs shows that some mismatches occur at the PPM1A‐NleD contact interface (Figure S6A). Both N‐ and C‐termini of NleD_EPEC_ are docked with PPM1A, while the N‐terminus of NleD_CR_ is disengaged from PPM1A (Figure S6B,C). This predicts a weaker interaction between NleD_CR_ and PPM1A. We then performed structural alignment of NleD_EPEC_ with NleD of *Salmonella arizona*, that share ∼78% identity with NleD_EPEC_. The alignment predicts that NleD_Sal_ should bind PPM1A similarly to NleD_EPEC_ (Figure S6A–C).

To test these model‐directed predictions, we expressed SBP‐tagged NleD_EPEC_, NleD_Sal,_ and NleD_CR_ and used them for capturing PPM1A expressed in HEK293T cells. As before, NleD_EPEC_ showed a robust interaction with PPM1A. NleD_Sal_ also bound PPM1A, but NleD_CR_ showed poor binding to PPM1A (Figure 5B). These results further validate the model of the NleD‐PPM1A complex. In addition, these data indicate that not all the members of the NleD family evolved to interact with PPM1A. The high similarity between the human and mice PPM1A (Figure S7), suggests that the NleD_CR_ would not bind also the essentially identical murine PPM1A and that this inability might reflect a difference in the infection strategies of EPEC and *C. rodentium*.

### 2.11 The Impact of NleD Proficiency in PPM1A Inhibition on Host Colonization

*C. rodentium* (CR) is a natural mouse pathogen that shares key colonization mechanisms with EPEC, including similar T3SS and effector sets [29]. It is thus commonly used as a murine homolog to model EPEC infection. The two *nleD* alleles of CR encode for identical NleD proteins that do not bind PPM1A (Figure 5B), excluding using the native CR NleD to test for the role of PPM1A inhibition upon experimental mouse infection. Thus, to test whether inhibition of PPM1A by NleD contributes to colonization of mouse intestines, we employed a CR mutant deleted of its two native *nleD* alleles (ΔΔ*nleD*) and complemented with either NleD_EPEC_ or NleD_EPEC‐E143A_, both proficient in PPM1A inhibition, with the latter also being deficient in MAPK cleavage (Figure 3). Complementation was performed using derivatives of the pGP706 suicide plasmid [30, 31], designed to integrate downstream of the genomic LEE6 operon by homologous recombination. This strategy places the integrated *nleD* alleles under control of the LEE6 promoter, restricting expression to infection conditions [21]. The experimental setup included a comparison of colonization levels of specific pathogen‐free (SPF) C57BL/6 mice infected with the following CR strains: wild‐type CR supplemented with an empty vector (YC11223 Table S1); CR ΔΔ*nleD* mutant supplemented with an empty vector (YS11228 Table S1); CR ΔΔ*nleD* mutant complemented with a vector expressing NleD_EPEC_ (YS11226 Table S1); and CR ΔΔ*nleD* mutant complemented with NleD_EPEC‐E143A_ (YS11227 Table S1). As an additional control, we used infection with wild‐type CR (ICC168 Table S1).

Mice were infected, and colonization efficiency was compared over time. The WT strains colonized the murine intestine and maintained a bacterial load higher than 10^8^ CFU/g faeces for up to 7 days post‐colonization (Figure 5C). In contrast, the ΔΔ*nleD* mutant exhibited attenuated colonization, with a 4‐log reduction in CFU/gr faeces, indicating that NleD is required for efficient host colonization (Figure 5C). We next tested the ΔΔ*nleD* mutants complemented with either *nleD*_EPEC_ or *nleD*_EPEC‐E143A_. Notably, at day 3 and 5, both *nleD*_EPEC_ and *nleD*_EPEC‐E143A_ partially restored host colonization (Figure 5C). This partial complementation suggests that NleD_CR_ may be a better fit for mouse infection. Notably, the NleD_EPEC‐E143A_ mutant's ability to partially restore colonization supports the role of its anti‐PPM1A activity in host colonization.

## 3 Discussion

We previously demonstrated that NleD from various pathogens specifically inactivates p38 and JNK, but not Erk, by cleaving within their TXY motif [15, 25]. Phosphorylation of the threonine (T) and tyrosine (Y) residues in this motif activates p38 and JNK. Here, we show that NleD fails to cleave the activated phospho‐p38/JNK. However, dephosphorylation of p38/JNK by PPM1A restores their susceptibility to cleavage by NleD. Surprisingly, in addition to its protease activity, NleD binds to and inhibits PPM1A. Structural analysis of the NleD‐PPM1A complex suggests that this inhibition occurs through steric hindrance caused by the bound NleD. These findings suggest that manipulation of host PPM1A might benefit enteropathogens such as EPEC, EHEC, and *Salmonella*.

### 3.1 The Seemingly Contradictory Functions of NleD

The rationale behind NleD's two seemingly counteracting functions: cleaving only unphosphorylated p38/JNK while simultaneously inhibiting their dephosphorylation by PPM1A, remains unclear. However, this paradox should be examined in the context of the coordinated actions of the full repertoire of effectors injected by EPEC into host cells [32]. Upon detecting attached EPEC, infected cells attempt to induce p38/JNK phosphorylation (Figure 1C) [14], but the injected NleE and NleD intercept this attempt. NleE blocks the activation of the MAP3K TAK1 [17, 18], thereby blocking downstream MAPK phosphorylation. Thus, by preventing p38/JNK phosphorylation, NleE synergizes with NleD, and reduces the necessity for PPM1A‐mediated dephosphorylation of the active p38/JNK. The biological significance of NleD's inhibition of PPM1A may extend beyond p38/JNK to other key PPM1A substrates, such as SMAD proteins [4] and AMP‐activated protein kinase α (AMPKα) [12].

### 3.2 The Mechanistic Basis for the Resistance of Phospho‐p38/JNK to NleD

The mechanistic basis for the resistance of phospho‐p38/JNK to NleD may be related to steric hindrance caused by the negatively charged phosphate groups on phospho‐p38/JNK, preventing NleD from accessing the TXY cleavage site. Alternatively, the phosphorylated activation loop may adopt a rigid conformation, rendering it resistant to NleD cleavage. This hypothesis aligns with our previous observation that NleD exhibits entropy‐dependent substrate specificity, cleaving MAPKs with flexible activation loops but failing to cleave the rigid activation loop of Erk, regardless of its phosphorylation state [25]. It is also possible that both the rigidity of the activation loop and the electrostatic repulsion from the phosphate groups contribute to resistance against NleD cleavage.

### 3.3 The NleD‐PPM1A Interaction

Analysis of the NleD‐PPM1A interaction yielded several novel insights. First, we show that NleD binds exclusively to N‐myristoylated PPM1A. Second, although PPM1A is traditionally classified as a serine/threonine phosphatase [12], we found that in vitro it effectively dephosphorylates both the threonine and tyrosine residues of the TXY motif in p38 and JNK, converting them into the unphosphorylated, NleD‐sensitive forms. This finding suggests that PPM1A should be redefined as a dual‐specificity p38 phosphatase. An additional unexpected discovery was that NleD inhibits PPM1A activity. This observation led us to investigate the mechanism of inhibition and its relevance to infection.

To understand how NleD inhibits PPM1A, we generated a model of the PPM1A‐NleD complex using in silico modeling, HDXMS interaction mapping, and cryo‐EM imaging. Our model suggests that NleD binds to PPM1A, sterically obstructing access to large substrates such as p38, thereby preventing dephosphorylation. The dependence of NleD‐PPM1A interaction on myristoylation remains unclear, as the myristoyl group is not visible in our structural model. Nonetheless, our model produced testable predictions, which we confirmed experimentally. Consistent with the model, we found that small substrates, such as pNPP, can still access the PPM1A catalytic site and be hydrolyzed. Additionally, as predicted, *Salmonella* NleD (NleD_Sal_) but not *C. rodentium* NleD (NleD_CR_) exhibited PPM1A binding. Of note, all these NleDs, similarly target p38 and JNK, but not ERK [25]. Our data suggest that NleDs from EPEC and *Salmonella*, but not *C. rodentium*, evolved to specifically interact with N‐myristoylated PPM1A and inhibit its activity.

### 3.4 How PPM1A Inhibition Might Benefit the Pathogen?

The absence of interaction between NleD_CR_ and PPM1A suggests that PPM1A inhibition may be maladaptive during *C. rodentium* infection. Consistent with this, complementation of *C. rodentium* lacking its endogenous NleDs (ΔΔnleD) with either NleD_EPEC_ or the catalytically inactive mutant (NleD_EPEC‐E143A_) resulted in only partial restoration of host colonization. The partial rescue by NleD_EPEC‐E143A_ indicates that NleD encodes proteolysis‐independent activities that can contribute to virulence, potentially including PPM1A inhibition. In contrast, the incomplete complementation by NleD_EPEC_, which retains both MAPK‐cleaving activity and PPM1A inhibition, suggests that effector functions advantageous in EPEC may be suboptimally balanced in the *C. rodentium* host context. It is unlikely that NleD_EPEC_ fails to interact with mouse PPM1A in these experiments, as human and mouse PPM1A share 98% sequence identity, and the few mismatches are located outside the PPM1A‐NleD interaction interface (Figure S7). Together, these findings support a model in which NleD activities are shaped by host‐specific selective pressures and require precise evolutionary tuning. Further mechanistic studies will be necessary to disentangle the relative contributions and potential interference of NleD's protease‐dependent and ‐independent functions during infection.

How PPM1A inhibition contributes to the virulence of EPEC and *Salmonella* strains remains unclear. It is conceivable that inhibition of the dephosphorylation of known PPM1A substrates benefits the pathogen. Reported substrates include p38, JNK, Erk, AMPKα, SMAD2/3 [3, 4, 12, 33], RelA/IKKβ [9], Yap/Taz [10], p62/SQSTM1 [11], CDK2/CDK6 [6, 7], CDK9 [5], as well as MAVS and TBK1 [8], with additional targets likely to be identified. Thus, inhibition of PPM1A would enhance phosphorylation‐dependent signaling across multiple pathways central for the intestinal epithelium hemostasis and immune response. Briefly, sustained phosphorylation of MAVS and TBK1, key mediators of viral RNA sensing, would enhance inflammation through type I interferon responses [8]. Inflammation might be further induced through RelA/IKKβ (i.e., NF‐κB activation) and p38/JNK hyper‐phosphorylation [9]. Prolonged phosphorylation of Smad2/3 would enhance TGF‐β signaling, which can impair renewal of the intestinal epithelium, compromise barrier integrity, to further boost the inflammation with pathological outcomes including fibrosis and increased risk of tumor progression [10, 33, 34, 35]. Persistent activation of CDK9 and CDK2/CDK6 might also increasing tumorigenic risk by accelerating G1/S progression, potentially driving uncontrolled epithelial proliferation [5, 6, 7]. In contrast, PPM1A dephosphorylates YAP/TAZ (e.g., YAP at S127), facilitating their nuclear localization and transcriptional programs that support intestinal regeneration; thus, inhibition of PPM1A and consequent YAP/TAZ cytoplasmic sequestration would impair regenerative capacity and delay epithelial repair [10]. Increased in phosphpo‐AMPKα may lead to chronic energy‐stress response with complex consequences [36]. Equally complex consequences are expected upon hyper‐phosphorylation of p62/SQSTM1 that may disrupt autophagic flux, leading to accumulation of damaged proteins and organelles, thereby compromising epithelial cell homeostasis [11]. Investigating whether NleD disrupts these pathways in vivo, and delineating the resulting consequences, represents an important direction for future research. It is noteworthy that NleD is injected by the T3SS alongside dozens of additional effectors, many of which target directly or indirectly the above host pathways and other pathways [13], adding another dimension to an already intricate and overlapping regulatory network.

In conclusion, this study underscores the complexity of bacterial effector function, highlighting that dual‐function effectors like NleD may be more common than previously recognized. It also emphasizes that the activity of individual effectors must be interpreted within the broader context of the full complement of effectors co‐delivered into host cells by the pathogen.

## 4 Methods

### 4.1 Cell Lines, Bacterial Strains, Plasmids, and Primers

Human embryonic kidney HEK293T cells (ATCC #CRL‐3216) were cultured in Dulbecco's Modified Eagle Medium (DMEM) with 10% fetal calf serum (FCS) and penicillin/streptomycin antibiotics (100 U/mL and 100 µg/mL, respectively). Expi293 cells (Thermo Fisher Scientific, A14635) were grown and used for expression using Expifectamine 293 medium according to the manufacturer's protocol.

Bacterial strains and plasmids used in this study are listed in Tables S1 and S2, respectively (Supporting Information data). Cloning was performed using either restriction enzymes or Gibson assembly. Inserts were amplified by PCR using the primers listed in Table S3. The primers and design of the suicide plasmids are described in Tables S2 and S3. Chromosomal integration was performed as previously described [21, 30, 31]

### 4.2 Transfections

Vector expressing PPM1A‐GFP fusion: HEK293T cells were plated in 35 mm dishes (0.5 × 10^6^ cells/well) and cultured in DMEM with 10% FCS and penicillin/streptomycin antibiotics for 18 h. Cells were transfected with 6 µg DNA per well using Turbofect (Thermo Scientific R0531) according to the manufacturer's protocol.

Vector expressing un‐tagged PPM1A: Expi293 cells in suspension were transfected using Expifectamine 293 (Thermo Fisher Scientific) according to the manufacturer's protocol. Expression was allowed for 72 h after transfection.

### 4.3 Infections

Cells seeded in 35 mm dishes were washed twice with antibiotic‐free DMEM and infected with 2 mL DMEM supplemented with 20 µL EPEC from an overnight standing culture at 37°C for the desired time (3 h unless mentioned otherwise). When mentioned, 25 ng/mL anisomycin or 20 ng/mL IL‐1 were added to the cells. After infection, cells were washed and lysed with RIPA buffer (Sigma R0278), centrifuged, and the supernatant was collected. The anisomycin used did not affect bacterial growth.

### 4.4 Protein Production in *E. coli* BL21

To produce SBP‐tagged NleD, the overnight culture of *E. coli* BL21 containing plasmids encoding the desired form of NleD was diluted 1:100 in LB supplemented with the appropriate antibiotics and grown for 2.5–3 h at 37°C to reach an OD_600_ of ∼0.6. The bacteria were then transferred to 16°C, and after 30 min, IPTG was added (0.2 mm). Expression was allowed for 18–20 h. The culture was centrifuged, and the pellet was resuspended in PBS supplemented with 150 mm NaCl, 0.1% Triton X‐100, 2 mm MgCl_2,_ and DNaseI. Bacteria were then lysed using a microfluidizer, and the lysate was cleared by centrifugation for 30 min at 20 000 RCF. Streptavidin‐agarose beads were used to capture NleD, and proteolysis and pull‐down assays were performed while the protein was bound to the beads.

To produce His‐tagged NleD, an overnight culture of *E. coli* BL21 containing a plasmid encoding NleD was diluted 1:100 in LB supplemented with the appropriate antibiotics and grown for 3 h at 37°C to reach an OD_600_ of ∼0.6. The culture was induced with 0.2 mm IPTG at 16°C for 18–20 h. To produce N‐myristoylated His‐tagged PPM1A, an overnight culture of *E. coli* BL21 containing plasmids expressing PPM1A and NMT was diluted 1:100 in LB supplemented with the appropriate antibiotics and grown for 3 h at 37°C to reach an OD_600_ of ∼0.6. The culture was induced with 0.2 mm IPTG at 16°C for 18–20 h. The pellets of NleD‐ or PPM1A‐ producing bacteria were resuspended in 50 mm Tris pH 8.0, 0.5 m NaCl, 5% glycerol, 10 mm β−mercaptoethanol (βME), and 0.1% Triton X‐100. The lysate was sonicated on ice for three cycles (1s ON, 2s OFF) at an amplitude of 29%. The lysate was centrifuged at 18 000 rpm (Beckman JA‐20) at 4°C for 30 min. The supernatant was subjected to Ni‐NTA beads for 1 h. After protein binding, the beads were washed three times with 20 mm imidazole and eluted twice with 0.3 and 0.5 m imidazole. The elution was desalted to Buffer A (50 mm Tris pH 8.0, 5% glycerol, 10 mm BME) and injected into a HiTrap Q HP column. The column was washed with 20 mL of Buffer A before applying a 0%–100% linear gradient with Buffer B (Buffer A + 1 m NaCl) to elute the protein. The protein of interest was injected to HiLoad 16/600 Superdex 75 pg pre‐equilibrated with 10 mm Tris pH 8.0 and 100 mm NaCl. The protein was flash frozen with liquid nitrogen and stored at ‐80°C.

For the production of p38 and P‐p38, we used overnight cultures of *E. coli* BL21 containing a plasmid expressing 6xHis‐tagged p38 alone, or co‐expressed with a constitutively active form of MKK6, a dual‐specificity MAP2K that phosphorylates both the T and Y residues of the p38 TXY motif [23]. Cultures were grown to an OD_600_ of ∼0.4 in LB, 37°C. The bacteria were then transferred to 30°C, and after 30 min, IPTG (1 mm) was added. Expression was allowed for 5 h. The culture was centrifuged, and the pellet was resuspended in buffer containing 50 mm Tris‐HCl pH 7.4, 0.5 m NaCl, 10 mm imidazole, 2 mm MgCl_2,_ and DNaseI. Lysis of the bacteria was performed using a microfluidizer. The lysate was cleared by centrifugation for 30 min at 20 000 RCF. Proteins were purified in an AKTA machine using a His‐trap column and eluted with an imidazole gradient up to 300 mm. Protein‐containing fractions were desalted and equilibrated with a buffer containing 12.5 mm HEPES pH 7.5, 100 mm KCl, and 1 mm dithiothreitol (DTT). For storage, glycerol was added to a final concentration of 6.25%. Purified proteins were stored at ‐80°C.

### 4.5 In Vitro Phosphorylation of p38

Purified p38α (20 µg) was incubated with purified constitutively active MKK6 in phosphorylation buffer (50 µm ATP, 20 mm MgCl_2_, 20 mm 2‐glycerolphosphate, 0.1 mm Na_3_Vo_4_, 1 mm DTT, 25 mm Hepes pH 7.5, final volume 40 µL) for 30 min at 30°C. 7.5 µg of the phosphorylated p38α was incubated at room‐temperature with 0.75 µg NleD in reaction buffer (50 mm Tris pH 8, 2 mm CaCl_2,_ and 50 mm NaCl, in a final volume of 20 µL). Reactions were stopped by adding Laemmli sample buffer (Bio‐rad #1610747).

### 4.6 Protein Production in HEK293T Cells

For the production of myristoylated PPM1A and its mutants, HEK293T were transfected with a plasmid expressing the desired 3xFLAG‐tagged version of PPM1A. Cells were seeded into 15 cm plates. The following day, transfection was performed: Plasmid DNA was diluted in 150 mm NaCl, and polyethylenimine (PEI) was added at a ratio of 4 µL PEI solution per 1 µg DNA. The tube was thoroughly vortexed and incubated for 30 min at room‐temperature to allow transfection complexes to form. The complexes were added to the cells, and after 5 h, the cells were washed twice, and the medium was changed to DMEM supplemented with 5% FCS. Expression was allowed for 72 h. Cells were scraped and resuspended in 20 mm Tris pH 7.4. A short sonication was performed, and then 150 mm NaCl and 1% Triton X‐100 were added. The lysate was cleared by centrifugation for 30 min at 20 000 RCF. Protein purification was performed on anti‐FLAG beads with elution using 3xFLAG peptide (Sigma) according to the manufacturer's protocol. 40% glycerol was added, and the protein was transferred to ‐20°C for long‐term storage. To preserve the enzymatic activity of PPM1A, the protein was never frozen before the enzymatic assay.

### 4.7 Protein–Protein Interaction (Pull‐Downs and Mass‐Spectrometry)

Lysates cleared as described above were incubated with streptavidin‐agarose (Sigma 51638) or glutathione‐agarose (Sigma 4510) beads for 1 h at 4°C, washed, and incubated for another 1 h with HeLa/HEK293 cell lysates or other bacteria lysates. After incubation, beads were washed and frozen for analysis by mass‐spectrometry or boiled in Laemmli sample buffer (Bio‐rad #1610747) for western blot analysis.

For MS analysis, the immobilized proteins were denatured, reduced, alkylated, and digested by standard procedures employing 8 m urea, dithiothreitol, iodoacetamide, and trypsin. Analysis of the resulting peptides was performed using a Q Exactive Plus mass spectrometer (Thermo Fisher Scientific, Waltham, MA USA) coupled online to a nanoflow UHPLC instrument, Ultimate 3000 Dionex (Thermo Fisher Scientific, Waltham, MA USA), using a 1%–80% acetonitrile gradient on a reverse phase 25 cm‐long C18 column (Thermo Scientific, PepMapRSLC). Mass spectra data were processed using the MaxQuant computational platform, version 1.5.3.12, and searched against translated coding sequences of the human proteome obtained from Uniprot. Relative protein quantification in MaxQuant was performed using the label‐free quantification (LFQ) algorithm.

### 4.8 Isothermal Titration Calorimetry (ITC)

The binding affinity between purified PPM1A and NleD was characterized using MircoCal PEAQ‐ITC. A total of 80 µm of NleD (350 mL) was used in the sample cell. Titrations were done using 750 µm of PPM1A. All the samples were thoroughly degassed and centrifuged to remove any precipitates. Binding measurements were performed at 25°C using 19 successive 2 µL injections (apart from the first injection), and separated by 150 s intervals to allow the peak to return to baseline. Binding measurements were set to maintain a reference power of 10 µcal s^−^^1^ and a stir speed of 750 rpm. Titration data were processed and fitted to a one‐site binding model using the MicroCal PEAQ‐ITC Analysis Software. Experiments were conducted three times.

### 4.9 In Vitro Proteolysis and Dephosphorylation Assays

Cleavage of p38 and JNK by NleD: SBP‐NleD was captured on streptavidin‐agarose beads as described above. The beads and p38 or P‐p38 were incubated for 1 h at 37°C in a buffer of 50 mm Tris pH 8.0, 50 mm NaCl and 2 mm CaCl_2_.

Assays of P‐p38 dephosphorylation by PPM1A were performed by the addition of the indicated purified proteins to a reaction buffer composed of 50 mm Tris, 50 mm Bis‐Tris, and 100 mm Acetate, pH 8.0. When needed, the reaction tube was incubated for 1 h at 4°C to allow protein binding and complex formation. The tubes were then transferred to 37°C, and the reaction was allowed for 1 h. In all cases, the reaction was stopped by boiling in the Laemmli sample buffer.

### 4.10 Amide Hydrogen Deuterium Exchange Mass Spectrometry (HDXMS)

To probe the protein‐protein interaction interface, hydrogen‐deuterium exchange mass spectrometry (HDXMS) was carried out for PPM1A and NleD proteins individually. The PPM1A‐NleD complex was generated by mixing the two proteins in a 1:2 stoichiometric ratio and incubating at 25°C for 30 min before HDX experiments. The deuterium labeling was initiated by diluting ∼100 pmol of each protein/complex in buffer prepared in deuterium oxide (final concentration ∼90% D_2_O) and incubating at 25°C for 1, 10, and 100 min time points. Non‐deuterated control experiments were performed by diluting the proteins in an aqueous buffer. The hydrogen‐deuterium exchange reactions were stopped using a chilled quench solution (1 m guanidinium‐HCl, trifluoroacetic acid) and then subjected to proteolysis by an online pepsin (Enzymate, Waters, USA) digestion. The quenched samples were pumped by flowing 0.1% formic acid in LCMS‐grade water at 100 µL min‐1 by an auxiliary solvent manager (M‐class ASM nanoACQUITY UPLC, Waters, USA). The digested peptides were then trapped (VanGuard C18 trap column) before being resolved by reverse‐phase chromatography (ACQUITY BEH C18, Waters, USA) using a 10 min gradient of 0.1% formic acid in acetonitrile (8%–40%), flowed at 40 µL per min, by M‐class binary solvent manager, maintained at 3°C (nanoACQUITY UPLC, Waters, USA) [37].

For detection and identification, the eluted peptides were electrospray ionized into a Synapt G2‐Si high‐resolution mass spectrometer (Waters, UK), operated in positive polarity. Ion‐mobility mode (HDMSe) was used for high‐resolution separation of the peptide ions, following the mass spectrometer parameters described previously [38]. Glu‐fibrinopeptide (m/z 785.8426, z = 2) was used as a lock mass solution, recorded at 30 s intervals. The mass spectra of peptides were identified using Protein Lynx Global Server 3.0.1, searched against a database of amino acid sequences of PPM1A and NleD proteins. The raw data from non‐deuterated controls were used to assign the peptide spectra, with identification parameters as described [39]. All HDXMS data were processed using DynamX HDX data analysis software version 3.0 (Waters, Milford, USA) with additional manual verification of the raw spectra. Only non‐overlapping peptides with a high signal‐to‐noise ratio were considered for final analysis. The deuterium uptake for individual peptides was calculated as the difference between the centroids of the isotopic distribution of the labeled peptide from the non‐deuterated peptide. Independent HDX measurements were carried out in triplicate, and the average data were used for final analysis and tabulated as Supporting Information files. HDX analysis resulted in 157 peptides covering ∼96% sequence of PPM1A, with an average redundancy of 4.9, while 141 peptides were observed for NleD, with a sequence coverage of 98% and an average redundancy of 7.3.

### 4.11 3D Modeling of PPM1A‐NleD_EPEC_ Complex Structure

Individual structures of full‐length PPM1A and NleD were modeled by Galaxy WEB (galaxy.seoklab.org) [40] and Robetta servers (robetta.bakerlab.org) [41]. The models from the two servers were consistent. The structure of hetero‐dimeric complex was predicted by the ClusPro server (cluspro.bu.edu). The model with the top score and consistent with HDX data was rigid‐fit in the cryo‐EM map by ChimeraX‐1.3.0. The catalytic sites of PPM1A were identified by superposing the crystal structure (PDB:4RA2) of the PPM1A monomer.

### 4.12 Preparation of Cryo‐EM Sample and Data Analysis

NleD was expressed with an N‐terminal MBP‐tag in *E. coli* strain BL21. PPM1A was expressed with a C‐terminal GFP‐strepII‐tag in *E. coli* strain BL21. NleD‐PPM1A complex was tandem‐affinity‐purified with dextran beads (BioBasic) and StreptactinXT beads (IBA), followed by gel‐filtration using Superdex75 16/600 (Cytiva). Au‐flat R1.2/1.3 grid (Protochips) was treated with 4 mm monothiolalkane(C11) PEG6‐OH (11‐mercaptoundecyl) hexaethyleneglycol (SensoPath Technologies) in ethanol, washed twice in ethanol, and air‐dried. 3 µL sample was applied on the grid, blotted, and plunge‐frozen in liquid ethane using FEI Vitrobot Mark IV. Images were collected by FEI Titian Krios at the Centre for Bio‐ Imaging Sciences, Singapore. The data were processed by Relion‐3.1.2 and cryoSPARC‐4.2.1. Overall resolution of the coulomb potential map was 5.3 Å.

### 4.13 pNPP Assay for PPM1A Activity

As described above, purified PPM1A and NleD were added to the reaction buffer and incubated for 1 h at 4°C to allow protein binding and complex formation. pNPP (1 mg/mL) was added, and the samples were transferred to 37°C. Cleavage of pNPP was assessed by serial absorbance measurements at 410 nm using a TECAN Spark 10 m plate reader. The results were plotted, and the slope was calculated to give the relative activity of PPM1A in each experimental condition.

### 4.14 SDS PAGE and Western Blot Analysis

Protein extracts were separated on Stain‐Free TGX gels (Bio‐Rad 456–8046) by SDS‐PAGE. Nitrocellulose membranes were probed by western blot analysis using the antibodies listed in Table S4. Western blots were normalized by adjusting total protein concentrations and validated through the Stain‐Free gels.

### 4.15 Experimental Infection

All animal experiments were approved by the Authority for Biological and Biomedical Models of the Hebrew University and conducted in accordance with International Animal Care and Use Committee (IACUC) guidelines under approval number NS‐22‐16836‐4. Mice were maintained under specific pathogen‐free (SPF) conditions in temperature‐controlled cages (22°C) with a 12 h light/dark cycle and ad libitum access to food and water, and were inspected regularly by SPF personnel. Infection experiments with *C. rodentium* were conducted in a biosafety level 2 facility, with daily monitoring for signs of illness and strict adherence to humane endpoints to minimize suffering.

5–8‐week‐old SPF C57BL/6J mice were pre‐treated with an intragastric dose of 2.5 mg vancomycin in 100uL DDW per animal, to reduce microbiota colonization resistance and facilitate pathogen colonization. 3 h later, mice were inoculated with 5 × 10^9^ CFU per mouse of a single *C. rodentium* strain suspended in 100 µL PBS by oral gavage. Bacterial load in faeces and dosage of bacterial inoculum were determined by serial dilutions of collected faeces and selective plating using MacConkey and tetracycline‐containing agar plates.

The C57BL/6 mice obtained from our supplier (Envigo) exhibit a high level of resistance to C. rodentium infection, likely due to vendor‐specific differences in the gut microbiota that confer strong colonization resistance, a phenomenon previously described [42, 43]. To overcome this barrier and ensure consistent establishment of infection, mice were given a single oral dose of vancomycin three days prior to inoculation. This treatment selectively reduces Gram‐positive members of the microbiota that contribute to colonization resistance, thereby creating a permissive environment for colonization of gram‐negative Enterobacteriaceae as described [44].

### 4.16 Statistical Analyses

Statistical analyses appropriate to each type of experiment are described in the corresponding sections in Methods and figure legends. Prism GraphPad or Microsoft Excel software was used to assess significance using an unpaired Student's *t*‐test. For the mouse colonization experiment, the geometric mean ± standard error are shown with individual data points plotted for each mouse to illustrate biological variability (*n* = 5 mice per group). Statistical significance was calculated using GraphPad Prism (GraphPad Software) with an unpaired, one‐tailed Student's *t*‐test. Exact *p*‐values are reported, with *p* < 0.05 considered statistically significant.

## Conflicts of Interest

The authors declare no conflicts of interest.

## Supporting information




**Supporting File 1**: advs75033‐sup‐0001‐SuppMat.docx.

**Supporting File 2**: advs75033‐sup‐0002‐Proteomics Supplemetary Table S5 concise.xlsx.

**Supporting File 3**: advs75033‐sup‐0003‐Data.xlsx.

## Data Availability

The data that support the findings of this study are available in the supplementary material of this article.
