# Supplementary material for: A Bifunctional T3SS‐Effector Simultaneously Cleaves Host MAP Kinase and Inhibits PPM1A Phosphatase
Source: Adv Sci (Weinh). 2026 Mar 28;13(37):e09702. doi: 10.1002/advs.202509702 (PMC13325838; doi:10.1002/advs.202509702)
Supplement: Supplementary file 1 — Supporting File 1: advs75033‐sup‐0001‐SuppMat.docx. [file ADVS-13-e09702-s001.docx]

**Supporting Information**

**A bifunctional T3SS-effector simultaneously cleaves host MAP kinase and inhibits PPM1A phosphatase**

Yaakov Socol^1*^, Lihi Gur-Arie^1*^, Netanel Tzarum^2^, Tamara Wellins^2^, Naama Katsowich^1^, Miriam Ravins^1^, Michal Bejerano-Sagie^1^, Klil Cohen^1^, Oded Livnah^3^, Joshua N Adkins^4^, Ernesto Nakayasu^4^, Alexei Savchenko^5^, Yeu Khai Choong^6^, Nikhil Kumar Tulsian^6,7^, Saturo Machida^6^, Sigal Ben-Yehuda^1^, Yael Litvak^2**^, J Sivaraman^6**^, Ilan Rosenshine^1**^

^1^Department of Microbiology and Molecular Genetics, Faculty of Medicine, The Hebrew University of Jerusalem.

^2^Department of Biological Chemistry, Alexander Silverman Institute of Life Sciences, The Hebrew University of Jerusalem, Jerusalem, Israel

^3^The Wolfson Center for Applied Structural Biology, The Edmond J. Safra Campus, The Hebrew University of Jerusalem, Jerusalem, Israel

^4^Biological Sciences Division, Pacific Northwest National Laboratory, Richland, Washington 99352, United States.

^5^Department of Microbiology, Immunology and Infectious Diseases, University of Calgary, Calgary, AB T2N 4N1, Canada.

^6^Department of Biological Sciences, 14 Science Drive 4, National University of Singapore, Singapore

^7^Department of Biochemistry, 28 Medical Drive 6, National University of Singapore, Singapore

**
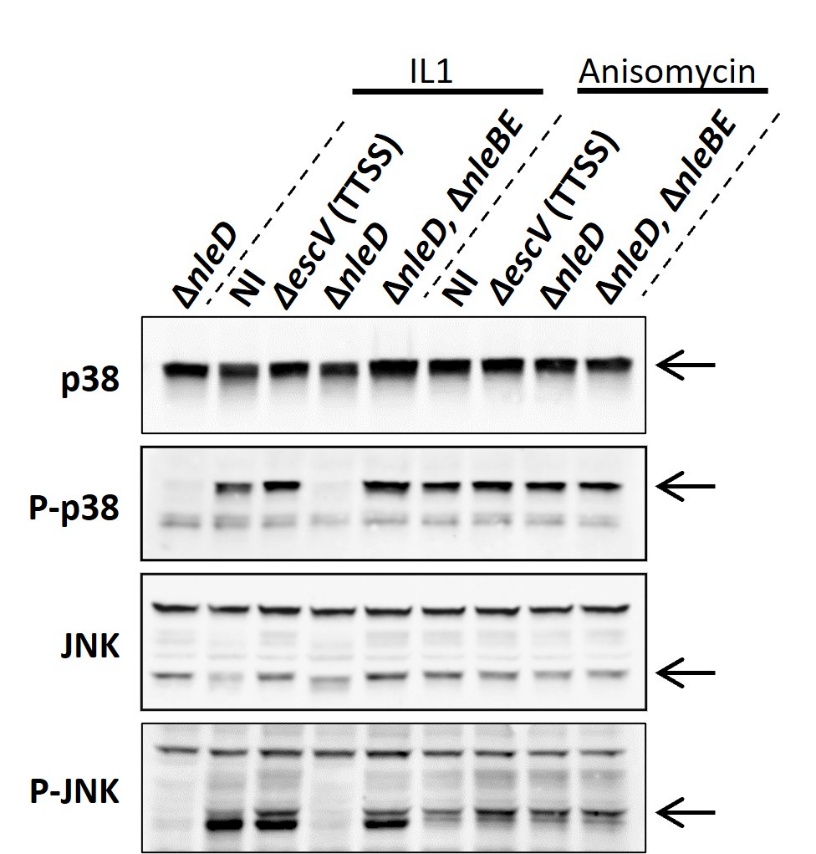
Supplementary figures**

**Supplementary Figure S1 NleE and NleD function in tandem to block JNK/p38 activation**

HeLa cells were infected with different EPEC strains for 3h, allowing them to inject effectors. Since cleavage of JNK and p38 by NleD would mask possible inhibition of their activation by NleE, we infected the cells using EPEC deleted of *nleD* (*ΔnleD*) or the double mutant *ΔnleD*, *ΔnleEB*. The *nleB* gene is proximal to *nleE* and was eliminated since it also interferes with NF-κB signaling. As additional controls, we included a mutant deficient in T3SS biogenesis (*ΔescV*) and uninfected cells (NI). We first allowed the bacteria to inject the host cells with the T3SS effectors. The cells were then treated with IL-1 or anisomycin, and the capacity of these treatments to induce JNK/p38 activation (i.e., their phosphorylation) was tested by Western blot using anti-phospho-JNK/p38 antibodies. JNK, p38 and their phospho forms are indicated by arrows. Notably, while IL-1 activates MAPK signaling through TAK1, anisomycin does so by activating MLK7 without requiring TAK1 (Figure 1A). The data show that the EPEC *ΔnleD* mutant, which has functional NleE, strongly inhibits JNK/p38 phosphorylation induced by IL-1 but not by anisomycin. In contrast, IL-1 treatment strongly induces JNK/p38 phosphorylation in cells infected with the EPEC *ΔnleD*, *ΔnleEB* double mutant. These results confirm that NleE inhibits JNK/p38 activation via the TAK1-dependent pathway and imply that NleE and NleD function in tandem to block JNK/p38 activation.

**
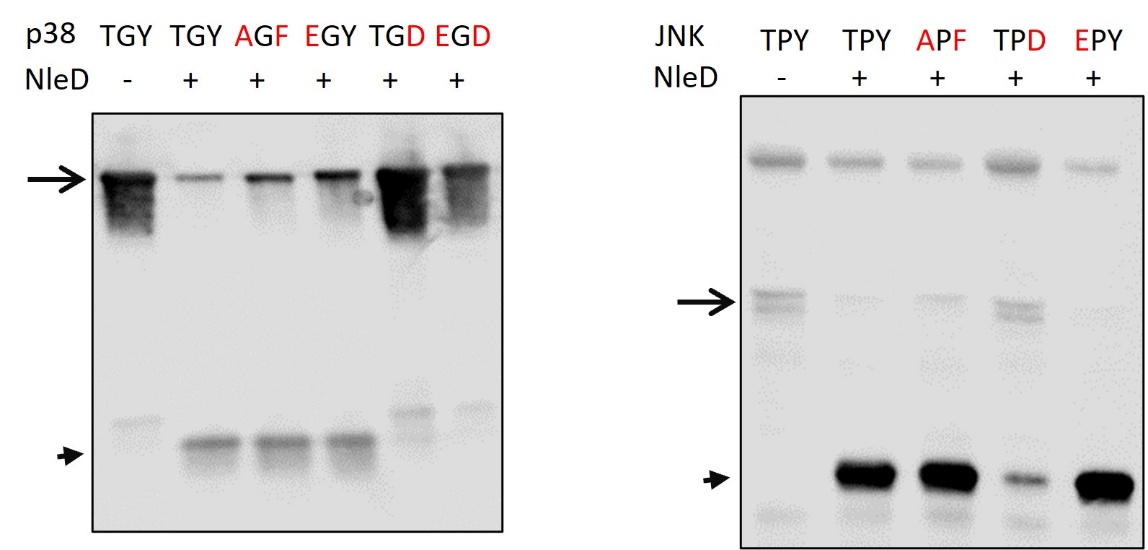
**

**Supplementary Figure S2. NleD fails to cleave p38 and JNK mutants where TXD replaced the TPY motif**

SBP-tagged NleD and His-tagged JNK or p38 variants were co-expressed in *E. coli* BL21 (Right and left panels, respectively). The JNK and p38 variants include different mutants in the TXY motif that are either neutral or negatively charged as indicated in red fonts. Proteins were extracted from the bacteria and subjected to Western blot analysis with an anti-His antibody. The cleavage products are indicated by arrowheads and the substrate by arrows.

**
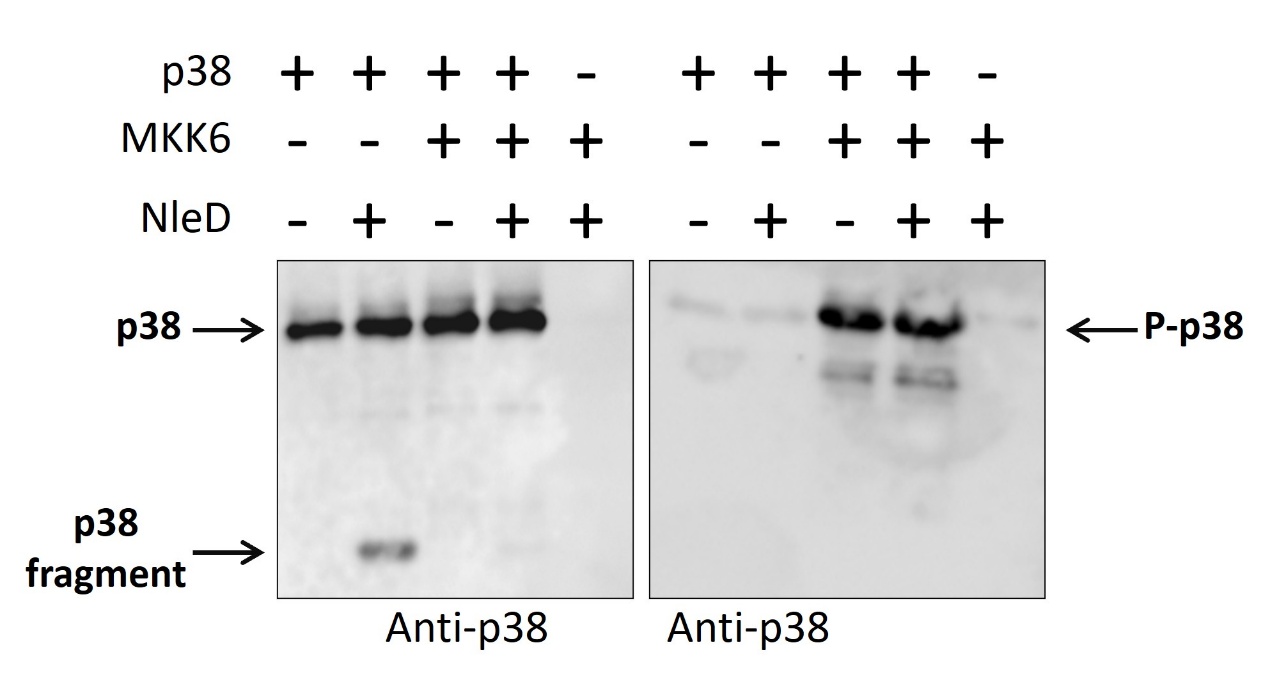
**

**Supplementary Figure S3. NleD fails to cleave phospho-p38 in vitro**

Purified NleD and p38 were incubated in the presence or absence of purified constitutively active MKK6 that phosphorylates p38. Intact and degraded p38 were detected using anti-p38 and anti-phospho-p38 (P-p38) antibodies.

**
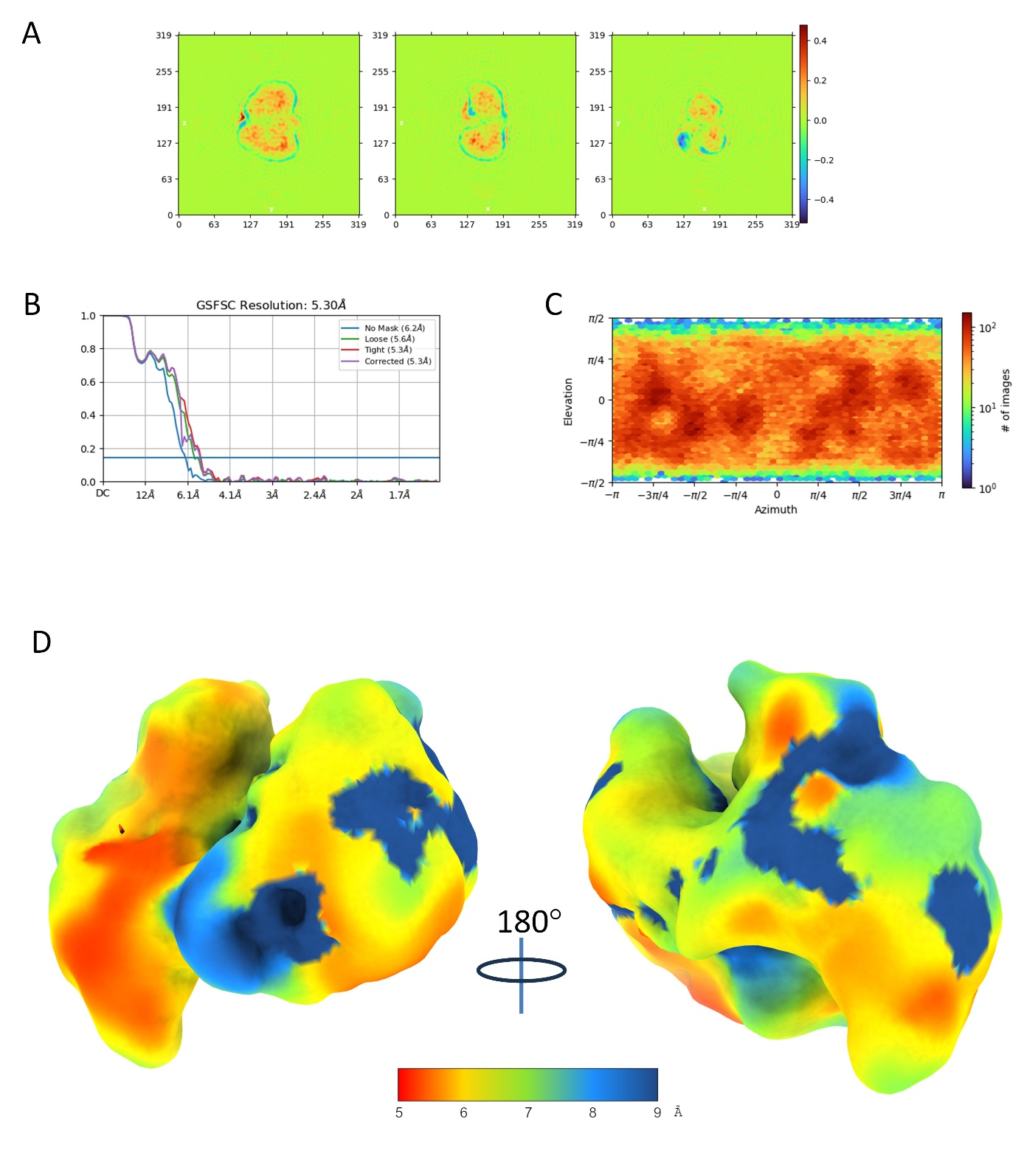
**

**Supplementary Figure S4. Single particle reconstruction of NleD-PPM1A complex.**

(A) Real space slices of Non-Uniform Refinement in CryoSPARC-4.2.1. (B) GSFSC curve of map resolution by FSC cut-off 0.143 after auto-tightening. (C) Orientation distribution at the final iteration. (D) Map coloured with local resolution by FSC cutoff 0.5.

**
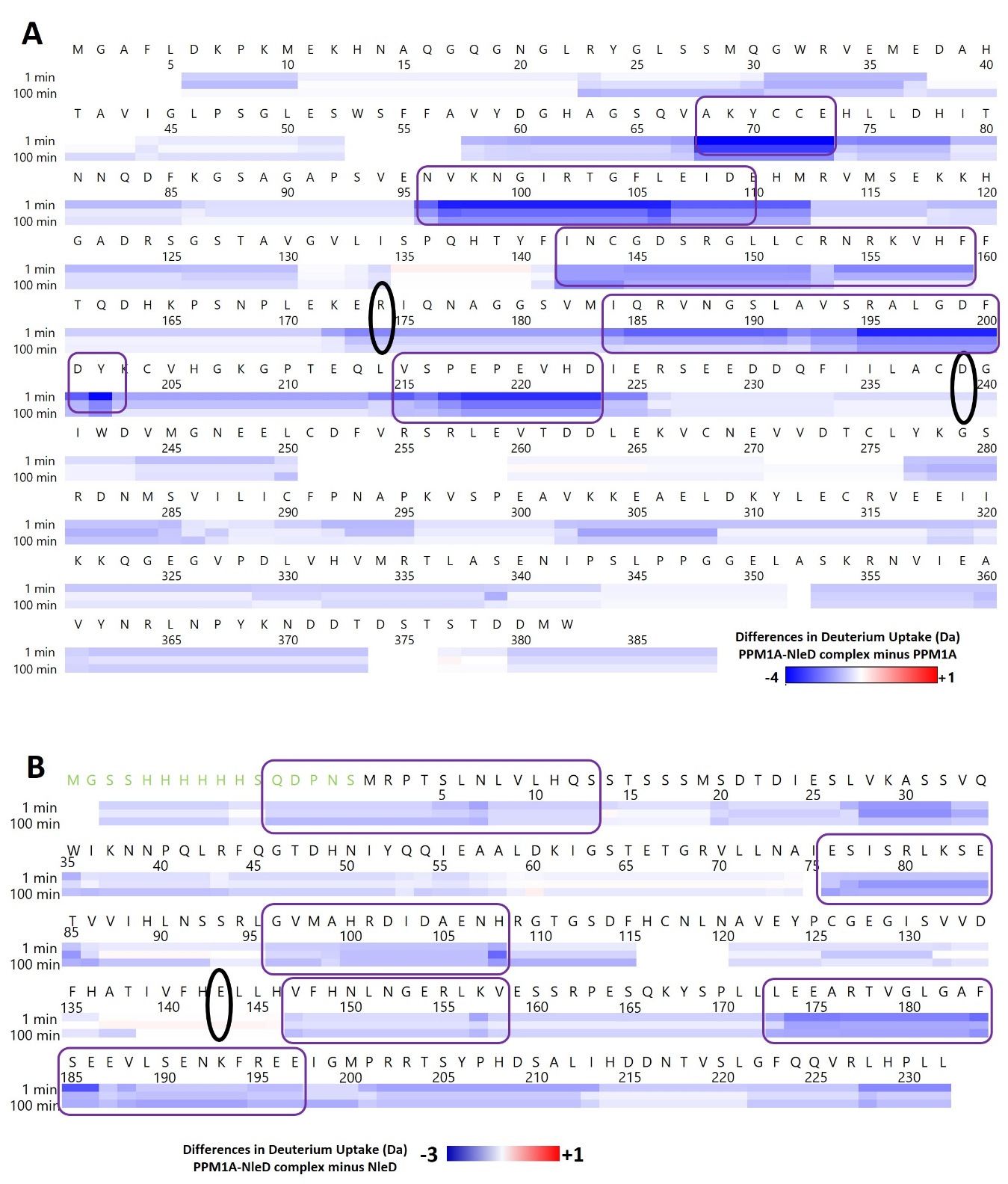
**

**Supplementary Figure S5**. **HDXMS results identify the sites of interaction between PPM1A and NleD.**

Differences in deuterium exchange of overlapping peptides were calculated to generate a heat map, which shows residues with most significant contribution (blue) involved in binding to PPM1A (panel A) and to NleD (panel B) upon complex formation. For PPM1A, residue stretches 68-73, 96-112, 142-159, 184-202 and 215-223 showed large-scale protection against deuterium uptake. Two residues, R174 and D239, which were mutated in this study are highlighted with black oval. For NleD, peptides 1-13, 76-86, 96-108, 147-158, 173-197 showed large-scale protection against deuterium uptake. Corresponding to the mutational analysis, E143 of NleD is highlighted with oval. The N-terminal residues of NleD in green (panel B) derive from the protein expression vector and are not counted.


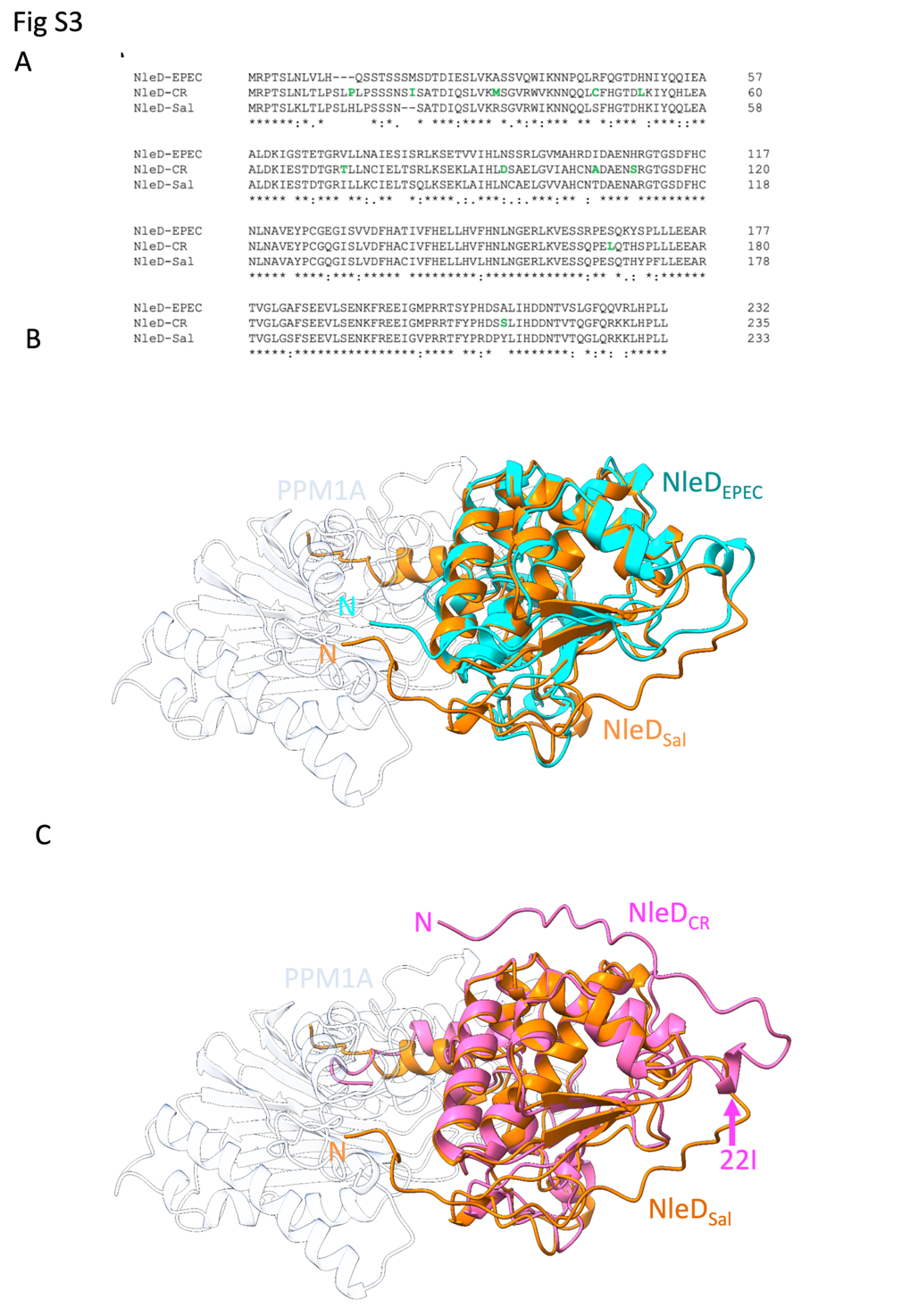


**Supplementary Figure S6. Structural comparison of EPEC, *Salmonella arizonae,* and *C rodentium* NleDs.**

(A) Amino acid sequence alignment of NleDs of EPEC, *C. rodentium*, and *S.* *arizonae* (NleD_EPEC_, NleD_CR_, NleD_Sal_). Green letters indicate residues unique to NleD_CR_. (B) NleD_Sal_ (orange) is docked with PPM1A (light blue, transparency 80) by superposing on NleD_EPEC_ (cyan) with the same orientation as in Figure 4G. (C) Structural alignment of NleD_Sal_ (orange) and NleD_CR_ (pink). Residue 22 indicated by arrow is unique to NleD_CR_ and changes the orientation of N-terminal loop of NleD_CR_.

**
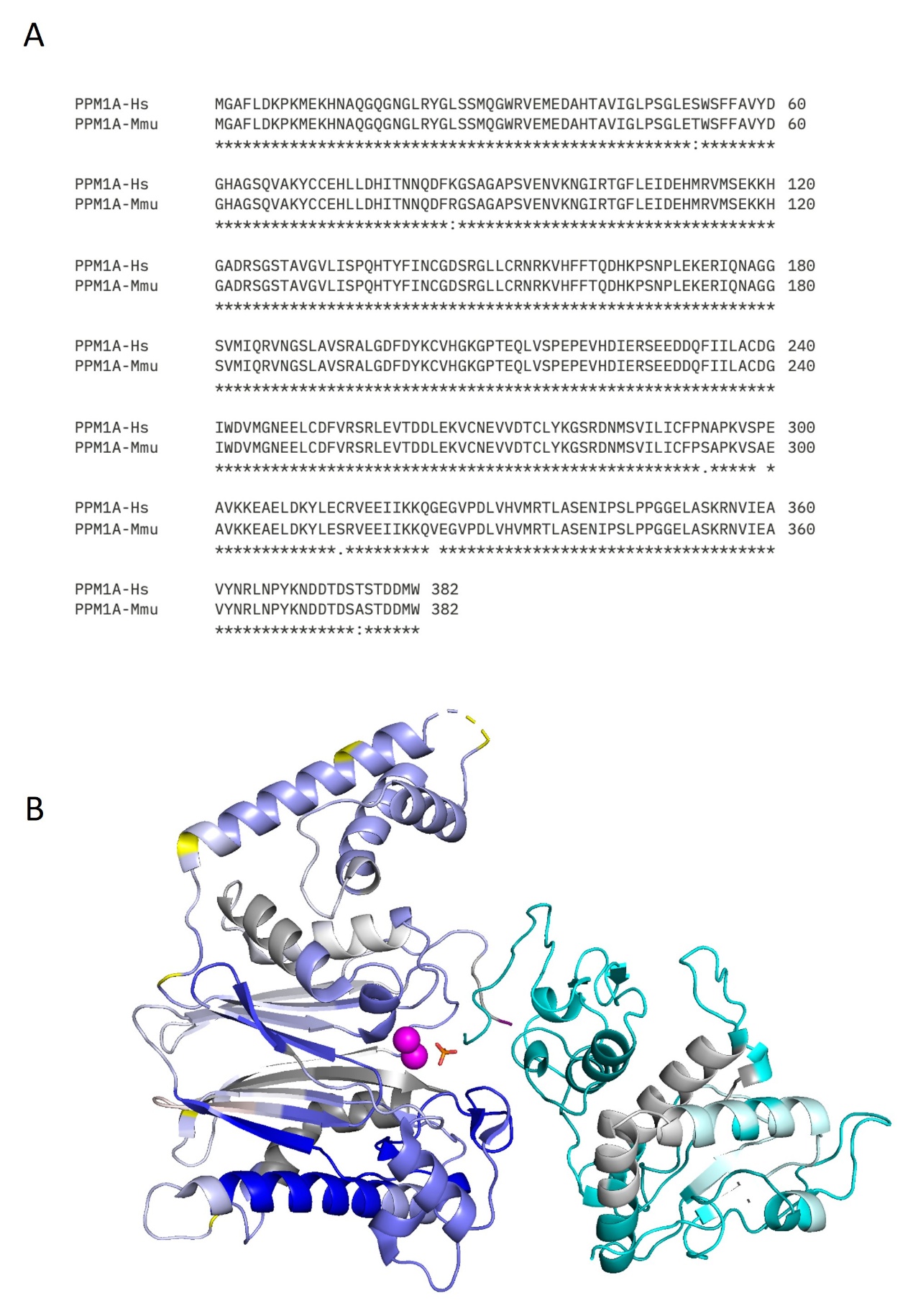
**

**Supplementary Figure S7. Human and mouse PPM1As**

(A) Clustal alignment of human (Hs) and mouse (Mmu) PPM1A sequences. Identities = 375/382 (98%), Positives = 378/382 (99%). Differences occur at residues 52, 86, 293, 299, 314, 326, 376. Human and mouse PPM1A shares 98 % sequences identity and have have exactly the same length

(B) A model of human PPM1A-NleD_EPEC_ complex as in Figure 4G. Residues 52, 86, 293, 299, 314, 326 in human PPM1A mismatching mouse PPM1A are highlighted in yellow. Residue 376 close to C-terminus is not modelled in crystal structure (PDB: 4RA2). These seven mismatches are located outside of PPM1A-NleD interaction interface indicating that probably the mouse PPM1A is equally able to bind NleD_EPEC_.

**Supplementary Tables**

**Supplementary Table S1**– Strains used in this study

| **Name** | **Description, genotype** | **Reference or source** |
| --- | --- | --- |
| E2348/69 | EPEC wild-type | J. Kaper |
| SN1961 | E2348/69 *escV*::Tn5*kan* | ^42^ |
| BL21(DE3) | F-, *ompT*, *hsdSβ*(rβ-mβ-), *dcm*, *gal*, (DE3) *tonA* | Novagen |
| SS6103 | EPEC *ΔnleD* scarless | This study |
| SS6107 | EPEC *ΔnleBE* scarless, *ΔnleD* scarless | This study |
| 9475 (ICC168) | CR wild type | ^43^ |
| YS11225 | 9475 containing *eae-*(insertion of pYS11222 empty vector) | This study |
| 9476 (ICC213) | CR Δn*leD1*,Δ*nleD2* (aka, ΔΔ*nleD*) | ^43^ |
| YS11228 | 9476*, eae-*(insertion of pYS11222 empty vector) | This study |
| YS11226 | 9476*,* eae-*nleD*_EPEC_ (insertion of pYS11220) | This study |
| YS11227 | 9476*,* eae-*nleD*_EPEC-E143A_ (insertion of pYS11221) | This study |
|  | E. coli K12 strain MC1061 lambda *pir* | Our collection |

**Supplementary Table S2** – Plasmids used in this study

| **Name** | **Description** | **Reference or source** |
| --- | --- | --- |
| pLG4470 | pET28 JNK2 | David Engelberg (HUJI) |
| pLG6391 | pET28 JNK2 where the TPY motif is replaced by APF | This study |
| pLG6428 | pET28 JNK2 where the TPY motif is replaced by TPD | This study |
| pLG6690 | pET28 JNK2 where the TPY motif is replaced by EPY | This study |
| pLG4821 | pET15b p38α | David Engelberg (HUJI) |
| pLG5041 | pET15b p38α where the TGY motif is replaced by AGF | David Engelberg (HUJI) |
| pLG6482 | pET15b p38α where the TGY motif is replaced by EGY | This study |
| pLG6487 | pET15b p38α where the TGY motif is replaced by TGD | This study |
| pLG5179 | pET15b p38α where the TGY motif is replaced by EGD | This study |
| pYS8540 | pACYC184-lacIq-MKK6(EE) | This study |
| 36943 | pET28 SBP TEV | Addgene |
| pLG5877 | pET28 SBP-*nleD* EPEC | This study |
| pLG5976 | pET28 SBP-*nleD* EPEC E143A | This study |
| pLG5878 | pET28 SBP-*nleD* *Salmonella* enterica ser. Arizona | This study |
| pLG5880 | pET28 SBP-*nleD* *Citrobacter rodentium* | This study |
| pLG6444 | pET16b hNMT | ^44^ |
| pEGFP_N1 | Expression vector | Clontech |
| pLG6283 | pEGFP-N1 hPPM1A fused to GFP | This study |
| pLG6323 | pEGFP-N1 hPPM1A_G2A_ fused to GFP | This study |
| pLG6626 | pEGFP-N1 PPM1A | This study |
| pSK7210 | pEGFP-N1 PPM1A R174G | This study |
| pSK7212 | pEGFP-N1 PPM1A D239N | This study |
| pYS8994 | pEGFP-N1 PPM1A-3xFLAG | This study |
| pYS8996 | pEGFP-N1 PPM1A D239N-3xFLAG | This study |
| pSA10 | pKK177-3 containing lacI^q^ | ^45^ |
| pLG6449 | pSA10 containing hPPM1A | This study |
| pYS11220 | A suicide plasmid based on pGP704 containing the last 500 bp of *C. rodentium* *eae* gene followed by RBS and *nleD*_EPEC_ | This study |
| pYS11221 | A suicide plasmid based on pGP704 containing the last 500 bp of C. *rodentium* *eae* gene followed by RBS and *nleD*_EPEC-E143A_ | This study |
| pYS11222 | A suicide plasmid based on pGP704 containing the last 500 bp of the *C. rodentium* eae gene followed by RBS, but not additional insert | This study |

**Supplementary Table S3** – Primers used in this study

| **Plasmid number** | **Primer sequence** |
| --- | --- |
| pLG6391 | GTGCACTAACTTCATGATGGCC CCTTTCGTGGTGACACGGTAC |
|  | GTACCGTGTCACCACGAAAGGGGCCATCATGAAGTTAGTGCAC |
| pLG6428 | GTGCACTAACTTCATGATGACACCTGACGTGGTGACACGGTAC |
|  | GTACCGTGTCACCACGTCAGGTGTCATCATGAAGTTAGTGCAC |
| pLG6690 | ATGGAACCTTACGTGGTGACACGG |
|  | CGTAAGGTTCCATCATGAAGTTAGTGCACGC |
| pLG6482 | CACACAGATGATGAAATGGAAGGCTACGTGGCCACTAGGTGGTAC |
|  | GTACCACCTAGTGGCCACGTAGCCTTCCATTTCATCATCTGTGTG |
| pLG6487 | CACACAGATGATGAAATGACAGGCGACGTGGCCACTAGGTGGTAC |
|  | GTACCACCTAGTGGCCACGTCGCCTGTCATTTCATCATCTGTGTG |
| pLG5179 | CACACAGATGATGAAATGGAAGGCGACGTGGCCACTAGGTGGTAC |
|  | GTACCACCTAGTGGCCACGTCGCCTTCCATTTCATCATCTGTGTG |
| pYS8540 | cttttgtaaaactgattcttggagactaaTAAGCTTATCGATGATAAGCTGTCAAAC |
|  | CTTATCGTCGTCATCCTTGTAATCCATAGTTAATTTCTCCTCTTTAATGAATTCTGTGTG |
|  | ATGGATTACAAGGATGACGACGATAAGATGGCGTCTCAGTCGAAAGGC |
|  | TTAGTCTCCAAGAATCAGTTTTACAAAAG |
| pLG5976 | GACTATTGTTTTTCATGCGTTGCTCCATGTTTTCC |
|  | GGAAAACATGGAGCAACGCATGAAAAACAATAGTC |
| pLG6283 | gcccttgctcaccatCCACATATCATCTGTTGATG |
|  | ccaccggtcgccaccATGGGAGCATTTTTAGACAAG |
|  | GGTGGCGACCGGTGGATC |
|  | ATGGTGAGCAAGGGCGAG |
| pLG6323 | gcccttgctcaccatCCACATATCATCTGTTGATG |
|  | ccaccggtcgccaccATGGCAGCATTTTTAGACAAG |
|  | GGTGGCGACCGGTGGATC |
|  | ATGGTGAGCAAGGGCGAG |
| pLG6626 | CATCAACAGATGATATGTGGTAAAGCGGCCGCGACTCTAG |
|  | CTAGAGTCGCGGCCGCTTTACCACATATCATCTGTTGATG |
| pSK7210 | CCAAGTAATCCGCTGGAGAAAGAAGGAATTCAGAATGCAGGTGGCTCTGTAATG |
|  | CTTCTTTCTCCAGCGGATTACTTGG |
| pSK7212 | GATCAGTTCATTATCCTTGCATGTAATGGTATCTGGGATGTTATGGGAAATG |
|  | ACATGCAAGGATAATGAACTGATCATC |
| pYS8994 | GATCATGATGGCGATTATAAAGATCATGATATTGATTATAAAGATGATGATGATAAATAAAGCGGCCGCGACTCTAG |
|  | CATGATCTTTATAATCGCCATCATGATCTTTATAATCCCACATATCATCTGTTGATGTAGAGTCAG |
| pLG6449 | cacaggaaacagaattcATGGGAGCATTTTTAGACAAG |
|  | tggctgcaggtcgacTTACTTGTACAGCTCGTC |
|  | GAATTCTGTTTCCTGTGTGAAATTGTTATC |
|  | GTCGACCTGCAGCCAAGC |

**Table S4** – Antibodies used in this study

| **Antibody** | **Company** | **Serial number** |
| --- | --- | --- |
| Anti-JNK | BD Pharmingen | 554285 |
| Anti-phospho-JNK | Cell signaling | 9251 |
| Anti-p38 | Santa Cruz | SC-535 |
| Anti-phospho-p38 | Cell signaling | 9216 |
| Anti-SBP | Santa Cruz | 101595 |
| Anti-6xHis | GE Healthcare | 27471001 |
| Anti-PPM1A | Cell Signaling | D18C10 XP |
| Anti-GFP | Clontech | 632460 |
